# AutoTube: a novel software for the automated morphometric analysis of vascular networks in tissues

**DOI:** 10.1007/s10456-018-9652-3

**Published:** 2018-10-28

**Authors:** Javier A. Montoya-Zegarra, Erica Russo, Peter Runge, Maria Jadhav, Ann-Helen Willrodt, Szymon Stoma, Simon F. Nørrelykke, Michael Detmar, Cornelia Halin

**Affiliations:** 1 Institute of Pharmaceutical Sciences, ETH Zürich, Vladimir-Prelog-Weg 1-5/10, 8093 Zurich, Switzerland; 2 Scientific Center for Optical and Electron Microscopy (ScopeM), ETH Zürich, Wolfgang-Pauli-Str. 14, 8093 Zurich, Switzerland

**Keywords:** Lymphatic vessels, Blood vessels, Quantification, Morphometric analysis, Whole-mounts, Tube formation

## Abstract

Due to their involvement in many physiologic and pathologic processes, there is a great interest in identifying new molecular pathways that mediate the formation and function of blood and lymphatic vessels. Vascular research increasingly involves the image-based analysis and quantification of vessel networks in tissue whole-mounts or of tube-like structures formed by cultured endothelial cells in vitro. While both types of experiments deliver important mechanistic insights into (lymph)angiogenic processes, the manual analysis and quantification of such experiments are typically labour-intensive and affected by inter-experimenter variability. To bypass these problems, we developed AutoTube, a new software that quantifies parameters like the area covered by vessels, vessel width, skeleton length and branching or crossing points of vascular networks in tissues and in in vitro assays. AutoTube is freely downloadable, comprises an intuitive graphical user interface and helps to perform otherwise highly time-consuming image analyses in a rapid, automated and reproducible manner. By analysing lymphatic and blood vascular networks in whole-mounts prepared from different tissues or from gene-targeted mice with known vascular abnormalities, we demonstrate the ability of AutoTube to determine vascular parameters in close agreement to the manual analyses and to identify statistically significant differences in vascular morphology in tissues and in vascular networks formed in in vitro assays.

**Electronic supplementary material:**

The online version of this article (10.1007/s10456-018-9652-3) contains supplementary material, which is available to authorized users.

## Introduction

Blood vessels and lymphatic vessels play important roles in diseases like cancer and inflammation [1–4]. Over the last decades, several signalling pathways that are important for the development or remodelling of the vasculature during pathologic conditions have been identified. Moreover, several anti-angiogenic drugs have been approved for the treatment of cancer and pathological ocular angiogenesis [5–7]. Traditionally, (lymph)angiogenic processes and the role of (lymph)angiogenic mediators on the vasculature have been analysed by investigating vascular parameters such as vessel density or vessel size in thin tissue sections stained by immunohistochemistry or immunofluorescence. However, a major limitation of these methods is that they only poorly reflect the complexity of the vascular network and do not allow assessing alterations in vascular branching or sprouting. In recent years, whole-mount immunofluorescent microscopy has therefore increasingly started to replace conventional staining of sections, particularly when working with murine tissues [8–12]. The advantage of the latter technique lies in its ability to capture the entire vasculature network in a two- or three-dimensional image. To characterise and quantify morphological differences of the blood and lymphatic vasculature in whole-mounts—for example in gene-targeted mice or upon treatment with pro-/anti(lymph)angiogenic mediators—various parameters, such as the area covered by vessels, the skeleton length, the vessel width and the number of branching or crossing points, are typically quantified in 2-dimensional (2D) projections of confocal image stacks. Additionally, to investigate and model (lymph)angiogenic processes in vitro, tube formation assays are frequently performed [13]. In these assays, the formation of vessel-like structures (tubes) by endothelial cells in gels containing extracellular matrix components is captured in 2D-microscopic images at a specific time point, followed by an image-based quantitative analysis of the vascular network (most importantly of tube length) [13, 14].

A major downside of these sophisticated methods to investigate (lymph)angiogenic processes in vivo and in vitro is the fact that the analysis of the resulting images is highly time-consuming. Moreover, the analysis is often biased by human subjectivity, resulting in considerable inter-experimenter variability. In the past, several pioneering studies have attempted to overcome these limitations and to automatically analyse the geometrical properties of vascular networks [15–20]. For example, image processing pipelines were developed by Guidolin et al. [16], Guidolin et al. [17] and Mezentsev et al. [19] that allowed for an automatic detection of vascular networks and also to extract geometrical measurements from images, such as branching points and their interconnections, lacunarities. Moreover, Mezentsev et al. [19] proposed a mathematical model that highlights the importance of the cell’s shape (round vs. elongated) during vascular development in in silico and in vitro studies. The topology of the vascular network is further measured in terms of number of branch points and the number of lacunae. In another work, Dhondt et al. [15] developed an online-closed framework to automatically segment and measure venation patterns in dark-field images. Moreover, Palm et al. [20] recently developed an efficient framework to simulate and to study the vascular network formation. Their model basically captures the self-organisation of endothelial cells into vascular network-like structures by considering cell shape information, cell adherence and their movements. All of the above-mentioned studies have undoubtedly reported promising new tools, which have helped to develop the field. However, a major factor that has limited their widespread use has been that—with the exception of the study by Palm et al. [20]—the source codes of these works are not available and the tools therefore cannot be extended or updated.

To bypass this issue, we developed a new and freely downloadable software tool called AutoTube, which allows for a robust, versatile and automated quantification of vascular network parameters in tissues as well in in vitro tube formation assays. Given that the source code of AutoTube is published online, the pipeline can be easily extended and adapted to the specific research question.

## Materials and methods

### AutoTube pipeline

**Fig. 1:**
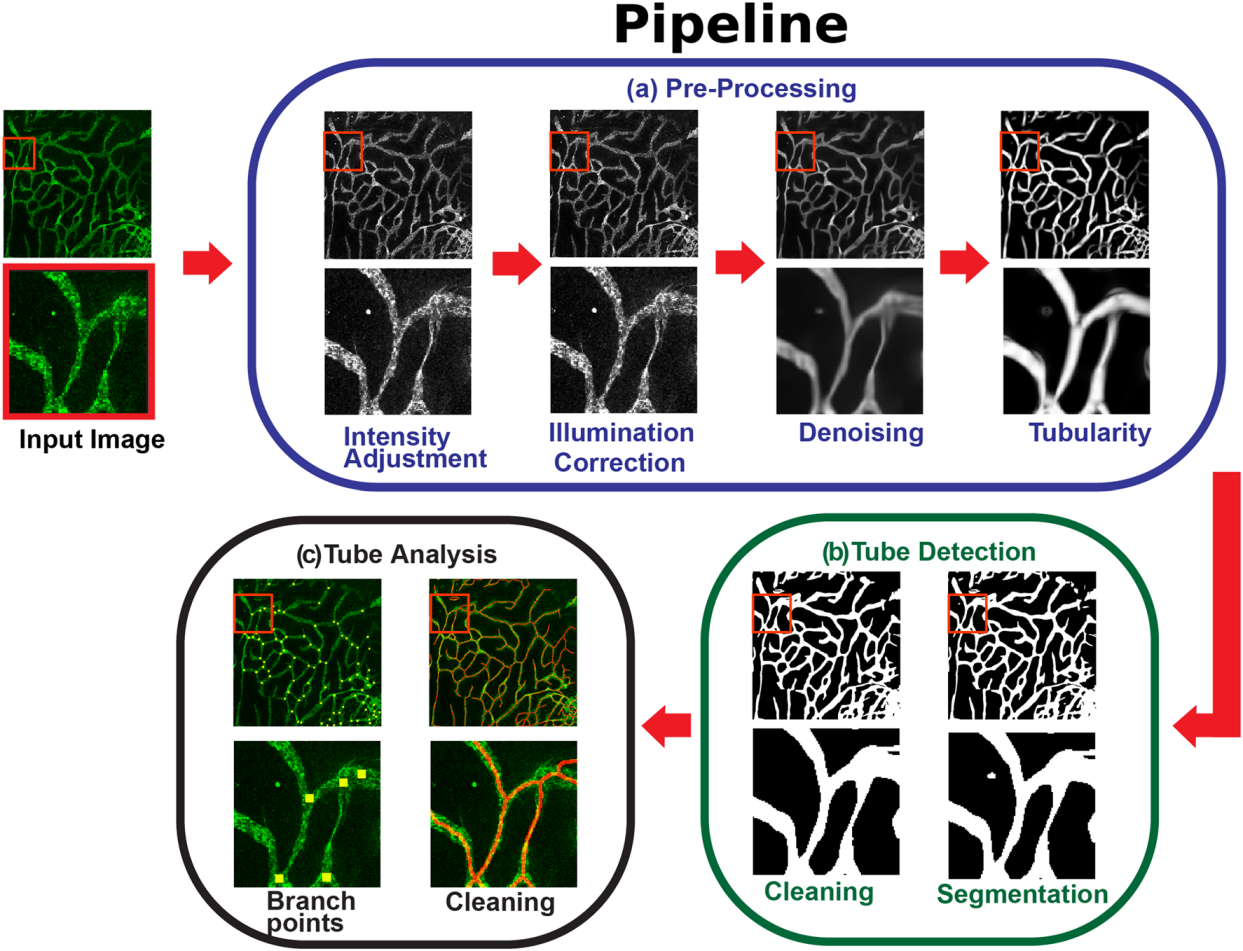
Overview of the AutoTube pipeline, which consists of three modules: **a** image pre-processing, **b** tube detection, and **c** tube analysis. In the *pre-processing* module, images are enhanced to compensate for detrimental image acquisition effects. These operations include image intensity adjustment, correction of uneven illumination and denoising. In the *tube detection* module, tubes are detected as foreground objects. Some image operations are also performed to refine the vessel detections. In the *tube analysis* module, a set of morphological measurements is extracted to quantify vessel properties, they include: the skeleton of the vessels and their associated branch points

A schematic overview of the AutoTube analysis pipeline is presented in Fig. 1. The pipeline is composed of three major modules, namely: (a) image pre-processing, (b) tube detection and (c) tube analysis.

In the *image pre-processing* module (Fig. 1a), input images are enhanced to reduce detrimental effects from image acquisition, such as poor contrast, uneven illumination and noise. One of the first steps for image enhancement consists of correcting the contrast of the images in such a way that it is easier to distinguish objects (e.g. vessels) from background. One key enhancement step consists in correcting uneven illumination. Unevenly illuminated microscopic images are characterised by spatially varying intensity, typically decreasing towards image edges. This is also known as vignetting [21] and typically can be attributed to different factors, such as the light path in the microscope [22]. Uncorrected uneven illuminated regions can negatively influence the segmentation step. It is also important to reduce the noise present in the images while preserving the finer details, to better facilitate the segmentation step.

In the subsequent *tube detection* module (Fig. 1b), vessels are detected directly in the enhanced images or after first finding tubular-like candidates using the Frangi Vesselness filter [23]. This latter step is especially useful when the staining is weak. Tube detection is done through image thresholding. As a result, a binary (black–white) mask is obtained in which vessels correspond to foreground objects (white regions) and all other regions are assigned to the background (black regions). In the AutoTube pipeline, a variety of thresholding techniques can be selected, depending on the quality of the stained images and on the characteristics of the dataset. For instance, if the input image is very noisy, a more conservative thresholding method such as the Otsu threshold should be used [24]. On the contrary, if the stained images are clean with good signal to noise ratio, a Kittler thresholding method is preferred [25]. The software can also remove small detected isolated regions which usually correspond to false-positive signals (e.g. caused by dirt or air-bubbles). The size of the isolated regions to be removed is adjusted by the user.

In the *tube analysis* module (Fig. 1c), the detected vessels are further analysed. Specifically, a set of morphology-based measurements are extracted from the detections. They include: (i) skeleton area, (ii) skeleton length, (iii) branching points, (iv) area covered by vessels. The pipeline allows the user to manually adjust the skeleton by pruning small skeleton branches or merging branch points that are spatially close to each other.

The software is available on GitHub under https://github.com/autotubularity/autotube. Moreover, a manual explaining the installation and step-by-step use of AutoTube can be found in the Electronic Supplementary Material.

The individual steps of the pipeline are explained in greater detail below.

### Image pre-processing (Fig. 1a)

Three different image pre-processing operations are considered in the first module (Fig. 1a), namely: (i) intensity adjustment, (ii) correction of uneven illumination and (iii) image denoising:

(i) *Intensity adjustment* the goal of image adjustment is to restore the contrast levels of the raw image to facilitate visual inspection and to normalise the image by using the full dynamic range. After image adjustment, the intensity range of the image is stretched. This means that after applying this pixel-wise operation, the available range of pixel intensity values is ideally fully covered (e.g. 0–255 for an 8-bit image). Three different methods can be selected in AutoTube for adjusting the image intensities, namely auto-contrast operation, histogram equalisation and adaptive histogram equalisation [26]. In the auto-contrast option, a fixed percentage of pixels at the low and high intensity ends are saturated. More precisely, two-pixel intensity values (p_low, p_high) are found at the low/high intensity ends in which all pixel values are smaller or greater than a fixed quantile (1% in our case). Pixel values outside (p_low, p_high) are mapped to the minimum/maximum values, and all intermediate values are assigned a value via linear interpolation. In histogram equalisation, the image intensity distributions (image histogram) of the original image are modified in such a way that the histogram of the output image matches approximately a uniform distribution. Finally, in the adaptive histogram equalisation, also known as Contrast-Limited Adaptive Histogram Equalisation (CLAHE [27]), a histogram equalisation normalisation is performed at image-patch level rather than at the entire image. This is especially useful, when the contrast across the different image regions is inhomogeneous. For all analyses performed in this study, the auto-contrast option was chosen (Table 1).
(ii) *Illumination correction* the correction of uneven illumination in images is done with the top-hat transform [28, 29]. More formally, the (white) top-hat transform is defined as the subtraction of the morphologically opened image from the original image. Given an image I and a circular structuring element *G*, the top-hat transform is defined as:
  $$TH(I)=I - (I*G),$$
  where the operator (*I* * *G*) is the morphological opening operator, i.e. an erosion followed by a dilation. We used a circular structuring element and a radial size of 51 pixels in all our experiments (Table 1). Intuitively, the top-hat transform can be seen as an operator that removes the image background and thus outputs an image with a background of uniform brightness. The morphological opening smoothens the images contours and eliminates small islands and sharp peaks [30]. Thus, after image subtraction all these finer details are kept and uneven illumination is corrected.
(iii) *Image denoising* an important step to restore images is denoising. It consists of reducing the noise present in images but at the same time preserving an object’s features such as edges, texture, etc. Our software provides two different options for image denoising: Wiener Filtering [31] and the Block-Matching and 3D filtering (BM3D) method [32, 33]. For all analyses performed in this study (Table 1), we have opted for using BM3D, since this method was previously shown in various datasets to be particularly suitable for preserving finer image structures [32, 33]. In BM3D, an image is first decomposed into groups of similar local patches to estimate the denoised image. Similar local patches are stacked together, and a 3D wavelet transform [34] is applied following a thresholding step, in which certain filter responses are set to zero. An inverse 3D wavelet transform is then applied, and the associated patches are combined to generate the estimated denoised image. In a second step, a Wiener collaborative filtering is applied to the original noisy image using the estimated denoised image as reference.

### Tube detection (Fig. 1b)

The goal of the vessel detection module is to partition the input image into foreground (vessels) and background-objects. After the pre-processing step, in which the input images are normalised, the images are converted into binary masks. For that purposes, four different thresholding approaches are available in our software, namely Otsu [24], Multi-Otsu [35], Kittler [25], and Adaptive [36]. In thresholding, objects that are brighter than the image background are detected as objects. Most of the thresholding methods rely on statistics computed over the image histogram. For our analysis, either Multi-Otsu or Kittler thresholding was performed (Table 1):

- *Otsu*/*Multi-Otsu* is amongst the most adopted thresholding techniques. The Otsu method assumes that the image contains two classes of pixels, i.e. foreground and background pixels [24]. The algorithm looks for the threshold that best minimises the variance within the two classes. An extension of the Otsu method is the Multi-Otsu method, in which instead of assuming only two modes, the number of modes is a parameter that is specified by the user (based on the expected number of pixel classes).
- *Kittler* the goal of the minimum-error thresholding method is to fit a combination of Gaussian distributions into the image histogram [25]. The general assumption is that the image pixels are generated from one of the two Gaussian distributions: the foreground distribution or the background distribution. In practice, the minimum-error thresholding method is able to detect weakly stained vessels. After image thresholding, a set of binary morphological operations are applied in order to refine the vessel detections. Specifically, small areas are removed and holes can also be filled.

### Tube analysis (Fig. 1c)

Once the foreground objects (vessels) are detected, the next step consists in extracting the vessel skeleton. To do so, an iterative image thinning operation based on the hit-and-miss transform is applied [31]. After each iteration, a set of n structuring elements pairs (J, K) are applied, one after the other, over a binary image B until no changes are seen. More formally, the thinning operation is defined as follows:

$$TH(I)=I - (I*G),$$

where the operator is equivalent to the subtraction of the hit-and-miss transform from the binary image *B*. The structuring elements pairs consist of 3 × 3 pixel neighbourhoods which delete pixels that have more than one foreground neighbour.

In essence, in the thinning operation, whenever there is an exact match between the foreground/background pixels in the structuring element and the foreground/background pixels in the region of the image under analysis, the region is set to background; otherwise, it is left unchanged. Once the skeleton is extracted, its associated branch points are computed by applying a set of 3 × 3 structuring elements that look for skeleton pixels having more than two adjacent points [37]. In addition, the user has the possibility to manually prune short skeleton ramifications and also to merge branching points based on minimal distance.

To prune short skeleton ramifications, starting from the skeleton endpoints, ramifications that are shorter than a specified length are removed. To do so, the user can specify in pixel units the length of the short ramifications to be removed in the *Spur length* parameter (Table 1). In addition, branch points lying close to each other within a selected circular distance are merged into one single branch point by averaging the spatial location of all the branch points falling within the selected radius. The circular distance is selected by the user in the *Spatial distance* parameter and is specified as pixel units (Table 1). In both cases, if the ramification length and the circular distance are set to zero, neither ramification pruning nor spatial branchpoint merging is applied.

### AutoTube parameters

For the analysis of the datasets presented in this study, the parameter settings reported in Table 1 were used. The individual steps and parameter/data entries needed for running AutoTube are further described in the User Manual (Supplemental Material). The entire image dataset used for generating Fig. 3 and Supplemental Fig. 2 has been deposited under the following link: 10.3929/ethz-b-000262426.

### AutoTube environmental settings

AutoTube is available as source code. We have run our experiments on a Windows 7 Professional Edition PC with Matlab R2016b. For image denoising, BM3D v2.0 (released on January 30, 2014) was used.^1^

### Mouse strains

Wild-type (WT) C57BL/6 mice were purchased from Janvier (Genest-Saint-Isle, France). Activated leukocyte cell adhesion molecule deficient mice (ALCAM^−/−^) [38], transgenic mice that overexpress ubiquitously the cytokine interleukin-7 (IL7tg) [39] and mice deficient for the IL-7 receptor α chain (IL-7Rα^−/−^) [40] were bred in our facility. All experiments were approved by the Cantonal Veterinary Office Zurich.

### Whole-mount immunofluorescence staining of ear skin and diaphragms

#### Lymphatic vessel detection

Ear skin and diaphragms were treated as previously described [9]. In brief, mice were euthanised and diaphragms and depilated ears were harvested. Ears were split into 2 halves along the cartilage, and the diaphragm was pinned on a self-made silica plate. Both tissues were fixed at room temperature for 2 h in 4% PFA (Merck, Readington, USA). The tissues were subsequently washed with 0.3% Triton-X/PBS (Honeywell, NJ, USA) and blocked in immunomix-1 (IM-1) (0.1% bovine serum albumin (BSA, Merck)/phosphate-buffered saline (PBS) and 5% normal donkey serum (Merck)). For lymphatic vessel detection, ears and diaphragms were then incubated overnight at 4 °C in IM-1 in the presence of 2 µg/ml rabbit anti-LYVE-1 antibody (1 µg/ml polyclonal, Angiobio, San Diego, USA).

#### Blood vessel detection

Mice were euthanised, and diaphragms and ears were harvested. The tissues were subsequently washed, blocked in immunomix-2 (IM-2) containing 0.1% Triton-X (Honeywell), 1% BSA/PBS (Merck) and 0.5% normal donkey serum (Merck). Both tissues were subsequently incubated overnight at 4 °C in IM-2 with rat anti-MECA-32 antibody (5 µg/ml, Biolegend, San Diego, USA).

The following day, the tissues were incubated for 3 h with Alexa Fluor-conjugated secondary antibodies, i.e. Alexa Fluor 594 anti-rat and Alexa Fluor 488 anti-rabbit (3 µg/ml, Invitrogen, Basel, Switzerland). Diaphragms were subsequently fixed in 4% PFA for 2 h at 4 °C. Samples were washed with PBS and mounted using Mowiol (Vector Laboratories, Burlingame, USA).

Tissues were analysed on a Zeiss LSM780 inverted confocal microscope (Carl Zeiss, Oberkochen, Germany) using a 10 × 0.3 NA EC Plan-Neofluar objective and processed with the Imaris software (version 7.1.1; Bitplane, Zurich, Switzerland). In the diaphragm, images of LYVE-1^+^ and MECA-32^+^ vessels were taken in the middle part of central tendon where the distribution of both lymphatic and blood vessels is homogenous and reproducible. In the ear skin, lymphatic and blood vessels were imaged at the external rim of the ear.

### In vitro tube formation assay

Human dermal lymphatic microvascular endothelial cells (LECs) (HMVEC-DLy, CC-2812, Lonza, Basel, Switzerland) were grown in tissue culture-treated dishes (TPP, Trasadingen, Switzerland) coated with 10 µg/ml collagen type I (Advanced BioMatrix, San Diego, CA, USA; 3.1 mg/ml). Cells were cultured in complete Endothelial Basal Medium-2 (EBM-2; Lonza, Walkersville, MD, USA) supplemented with 5% fetal calf serum (FCS, Gibco, Paisley, UK), 0.4% ml human basic fibroblast growth factor-β (bFGF), 0.1% human epidermal growth factor (EGF), 0.1% R3-insulin-like growth factor (IGF), 0.04% hydrocortisone, 0.1% ascorbic acid, 0.1% gentamycin/amphotericin-B (all from Lonza: EGM-2MV supplements) at 37 °C in humidified air with 5% CO_2_. For tube formation experiments, 40’000 LECs/well were seeded into a 24 well tissue culture-treated plate (TPP) previously coated with 10 µg/ml collagen type I (Advanced BioMatrix) and were incubated in complete EBM-2 medium (Lonza) at 37 °C. At confluency (usually after 2–3 days), LEC monolayers were washed with sterile PBS and cultured for 24 h in EBM-2 medium (Lonza) supplemented with 2% FCS (Gibco) and 1% antibiotic antimycotic solution (1×; Fluka, Buchs, Switzerland) (EBM-2 starvation medium). On the day of the experiment, a collagen stock solution containing 7 ml PureCol (3.1 mg/ml, Advanced BioMatrix), 0.8 ml sterile 10× PBS (pH 7.2, Gibco), 0.28 ml 0.1 M sterile NaOH (sodium hydroxide, puriss., diluted in sterile deionised water, Fluka) and 4.2 µl 10M NaOH (diluted in sterile deionised water, Fluka) was prepared and a physiological pH of 7.4 was confirmed. Subsequently, the collagen stock solution was diluted in EBM-2 starvation medium to a final concentration of 1 mg/ml, and cell monolayers were overlaid with 0.4 ml of 1 mg/ml collagen solution containing 20 ng/ml VEGF-A (Peprotech, London, UK) or containing sterile PBS as a control. Every condition was prepared in triplicates. Plates were incubated at 37 °C, and tube formation in the collagen gels was analysed after 14 h. To visualise the formed tubes, each well was incubated with 100 µl of 2.5 µM Cell Tracker Green CMFDA dye (Invitrogen) diluted in EBM-2 starvation medium (Lonza) (final concentration: 0.5 µM CMFDA per well) for 30 min at 37 °C. Representative images of tubes (3 images/well) were acquired on a fluorescent microscope (Nikon Eclipse Ti-E, Tokio, Japan) using a 4× objective and 1.5 zoom.

### Manual analyses of vessels in whole-mounts and in vitro tube formation assays

Manual image analyses of the total LYVE-1^+^ lymphatic and MECA-32^+^ blood vessel area were performed using Fiji [41]. The total vessel and tube length was determined with a self-made length-calculating macro using Fiji [41]. The average vessel width was calculated by dividing the area covered by vessels, measured in one particular image, by the length of the entire vascular network of the image. The number of branching points in whole-mount images and tube formation were quantified manually using Photoshop (Adobe CS6, San Josè, USA) (Supplementary Fig. 1). All manual analyses were performed in a blinded manner (i.e. with encrypted image file names that did not disclose the genotype or treatment condition).

### Statistical analysis

Results are presented as mean ± standard error on the mean (SEM). Datasets were analysed using the Student t test, and correlation was analysed using linear regression represented as *R*^2^. Differences were considered statistically significant when *p* < 0.05. Statistical analysis was performed with Prism 7 (GraphPad, La Jolla, CA, USA).

## Results

### AutoTube

The image analysis toolbox of AutoTube is available as a graphical user interface (GUI) and has been developed in the MATLAB (The Mathworks, Natick, MA, USA) numerical programming environment. The software is available on GitHub under https://github.com/autotubularity/autotube. Moreover, a manual explaining the installation and step-by-step use of AutoTube can be found in the Electronic Supplementary Material.

**Fig. 2:**
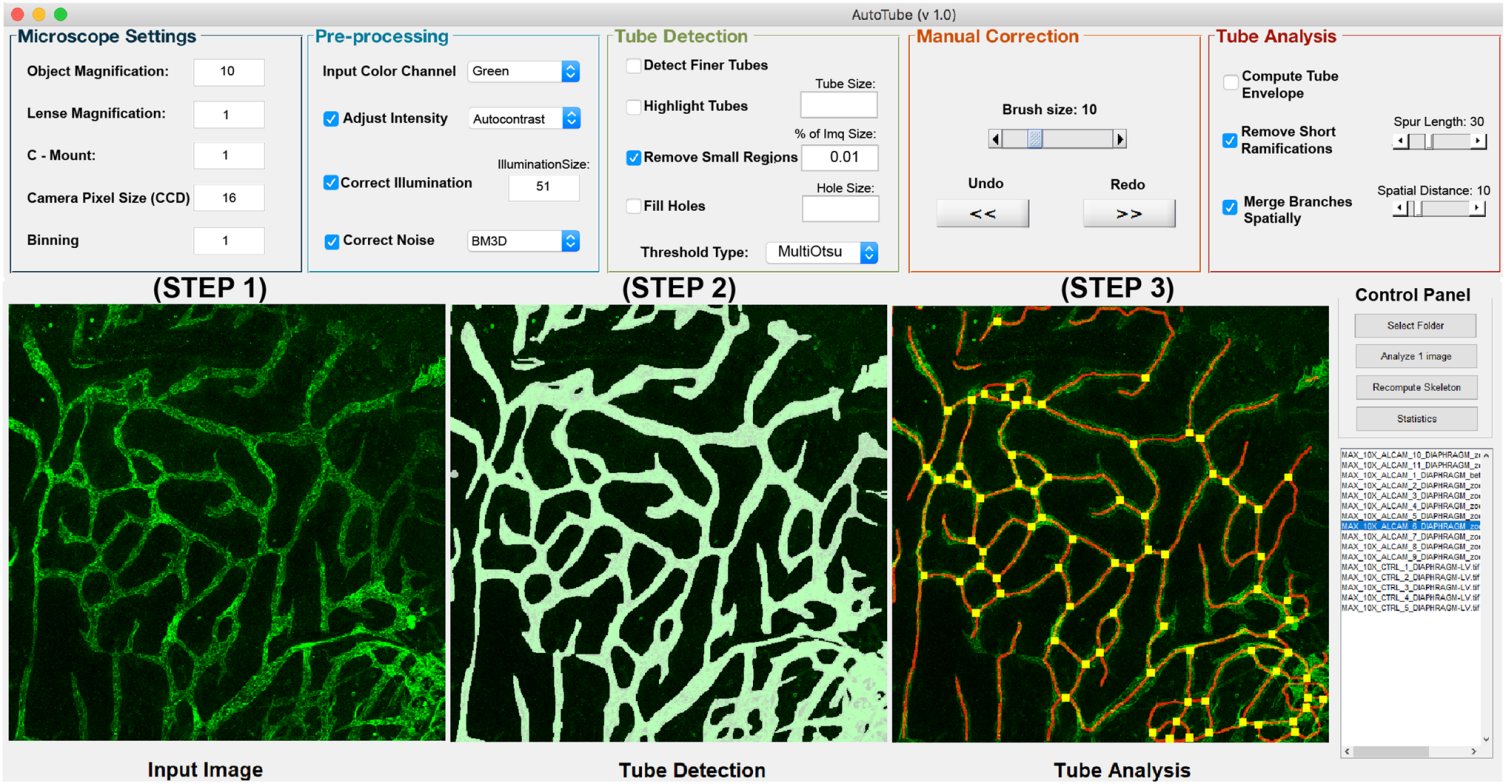
Graphical user interface (GUI) of AutoTube. The user first selects the input directory containing the images to be analysed **(STEP 1)**. Once this is done, the filenames of all images contained in the selected directory are displayed. Next, the user may adjust the image processing parameters or may use the default parameter values **(STEP 3)**. Once the user clicks on the “Analyze” button, the whole image processing steps are applied for each image and the intermediate results are displayed **(STEP 3)** and stored. Once the processing of all images is completed, an Excel file containing the image statistics is generated, after clicking on the “Statistics” button

The GUI (Fig. 2) enables the user to automatically analyse whole-mount stainings or in vitro tube formation images and adjust the software’s parameters accordingly. Specifically, AutoTube offers a set of customisable parameters that can be adapted to account for differences in image quality. The morphometric properties analysed include the area covered by vessels, the length of the vascular network and the numbers of branching/crossing points. A fourth parameter, namely the average vessel width, is calculated for each image by dividing the area covered by vessels by the length of the vascular network. Once an analysis is completed, a set of output directories is automatically created at the same location where the input images were found. For each intermediate step of the pipeline, an output image is separately stored. The latter is intended to help the user to adjust their parameters, better understand the analysis, and also to export the output images for reporting results. In addition, an Excel file containing the morphological measurements is automatically created and can be used to generate further summary statistics or plots (see Fig. 2 and User Manual in the Appendix). Before using AutoTube, the parameter settings that are best suited for the analysis of a particular dataset need to be determined. This involves first performing a manual analysis of the respective dataset and then running AutoTube with various parameter combinations to identify the one that best reflects the results obtained by the manual analysis. The parameters used by us for the different type of datasets described in this study are summarised in Table 1.

**Table 1:** Settings used for the acquisition and analysis of lymphatics and blood vessels in tissue whole-mounts and of in vitro tube formation datasets

	Lymphatic vessels	Blood vessels	In vitro tube formation
0. Microscope settings			
Microscope objective power	10 ×	10 ×	4 ×
Lens magnification	1 ×	1 ×	1 ×
C-mount	1 ×	1 ×	1 ×
Camera pixel size (µm)	2.76	0.758	6.5
Binning	1 (No binning)	1 (No binning)	1 (No binning)
Image resolution	512 × 512	1024 × 1024	2048 × 2048
1. Pre-processing			
Input colour channel	Green	Red	Green
Adjust intensity	Auto-contrast	Auto-contrast	Auto-contrast
Correct illumination	51	51	51
Image denoising	BM3D	BM3D	BM3D
2. Tube detection			
Detect finer tubes	Not selected	Not selected	Selected
Remove small regions	0.01	0.01	0.01
Fill holes			
Threshold type	Multi-Otsu	Multi-Otsu	Kittler
3. Tube analysis			
Spur length (pixel)	15	15	30
Spatial distance: (pixel)	10	10	10

### AutoTube analyses lymphatic networks in an accurate and reproducible fashion

To validate our software for the analysis of vascular networks in tissue whole-mounts, we analysed three genetic mouse models, for which our group has previously reported a lymphangiogenic phenotype [9]. Specifically, we investigated the ability of AutoTube to detect morphologic differences in the lymphatic vasculature of IL-7tg and IL-7Rα^−/−^ mice [9], and of ALCAM^−/−^ mice [42]. To this end, we stained ear skin and diaphragms of these gene-targeted mice and of WT controls for the lymphatic specific marker LYVE-1, to produce whole-mount data for side-by-side manual and automated analyses. For the manual annotation, two investigators independently analysed whole-mount images in a blinded manner. This involved ImageJ- or Photoshop-assisted processing of the images and manually measuring the lymphatic vessel area, length and the numbers of branching points (Supplementary Fig. 1). As with AutoTube software, the average lymphatic vessel width was calculated by dividing the lymphatic area by the lymphatic length.

**Fig. 3:**
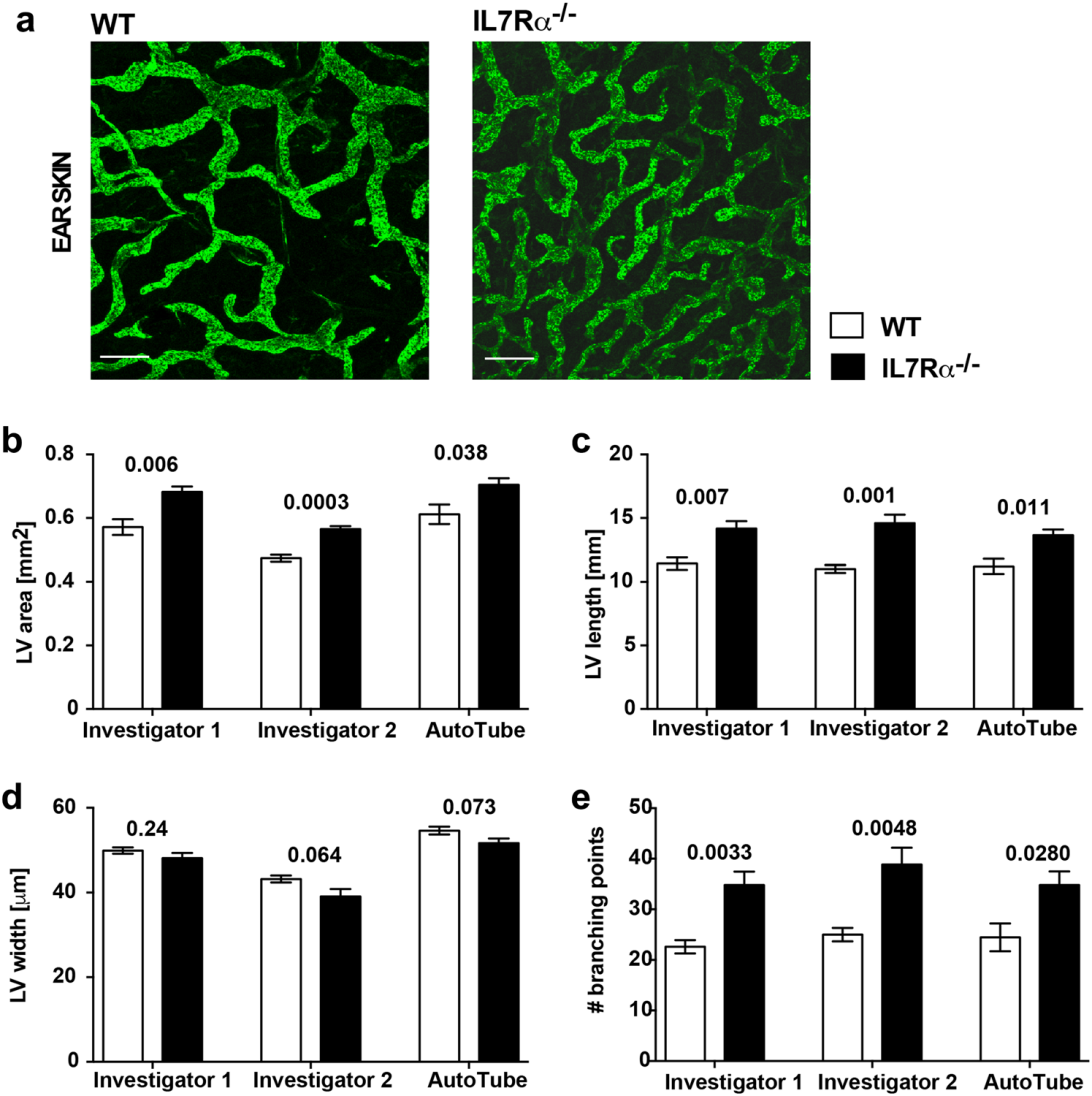
AutoTube-based and manual analysis of the LYVE-1^+^ lymphatic vasculature in murine ear skin. Parameters analysed include the area covered by (**b**) lymphatic vessels (LV area), (**c**) the absolute length of the skeleton (LV length), (**d**) the average vessel width (LV width) and (**e**) the number of branching points (# branching points) per picture analysed. (**a**) Representative images showing LYVE-1^+^ lymphatic vessels in the ear skin of WT and IL-7Rα^−/−^ mice. Scale bar: 100 µm. **b**–**e** Results reveal the morphologic differences in the dermal lymphatic network of IL7Rα^−/−^ mouse ear skin compared to WT mice. The data shown represent the mean ± SEM; statistical analysis was performed with Student’s *t* test; *n* = 5 mice each group (3 pictures/mouse). The AutoTube settings used are described in Table 1

To investigate the accuracy of the AutoTube software and its ability to compute lymphatic morphometric parameters, we first performed a side-by-side comparison of the measurements obtained by two different manual investigators and by the software, using the IL-7Rα^−/−^ dataset as an example (Fig. 3a). The two manual analyses as well as the AutoTube-based analysis yielded very similar results, namely showing a significant increase in the LV area, LV length and in branching points in IL-7Rα^−/−^ as compared to WT mice (Fig. 3a–e). Notably, the magnitudes of the responses observed were rather similar (Fig. 3b–e), but certain differences in the absolute values of the parameters analysed were observed, particularly in the measurements of the lymphatic vessel area and width (Fig. 3b, d). To better illustrate how the choice of parameters in AutoTube affects the results, we repeated the analysis of the same image dataset (Fig. 3a–e) using slightly different parameter settings (Supplementary Fig. 2). Specifically, we compared the impact of the Kittler and the Multi-Otsu thresholding methods, which both should be able to detect vessels even under suboptimal (weak) staining conditions. Additionally, we studied the impact of removing small detected vessel fragments, which often contribute to noise (false-positive signals), by either removing vessels smaller than 1% or smaller than 10% of the total number of pixels in the image (Supplementary Fig. 2). When comparing the impact of the various settings on our measurements, we found that the relative differences in lymphatic vessel area, width, and length and in branching point numbers were largely preserved regardless of the settings used (Fig. 3 and Supplementary Fig. 2). In the case of the lymphatic vessel area and length and the branching points numbers, typically higher absolute values were measured when selecting Kittler, likely because this thresholding method is able to detect more vessels than Multi-Otsu. Overall, these findings show that the results measured are robust and indicate that the choice of the thresholding method has a greater impact on the results than the setting of the post-processing parameters, like the removal of small detected vessels.

**Fig. 4:**
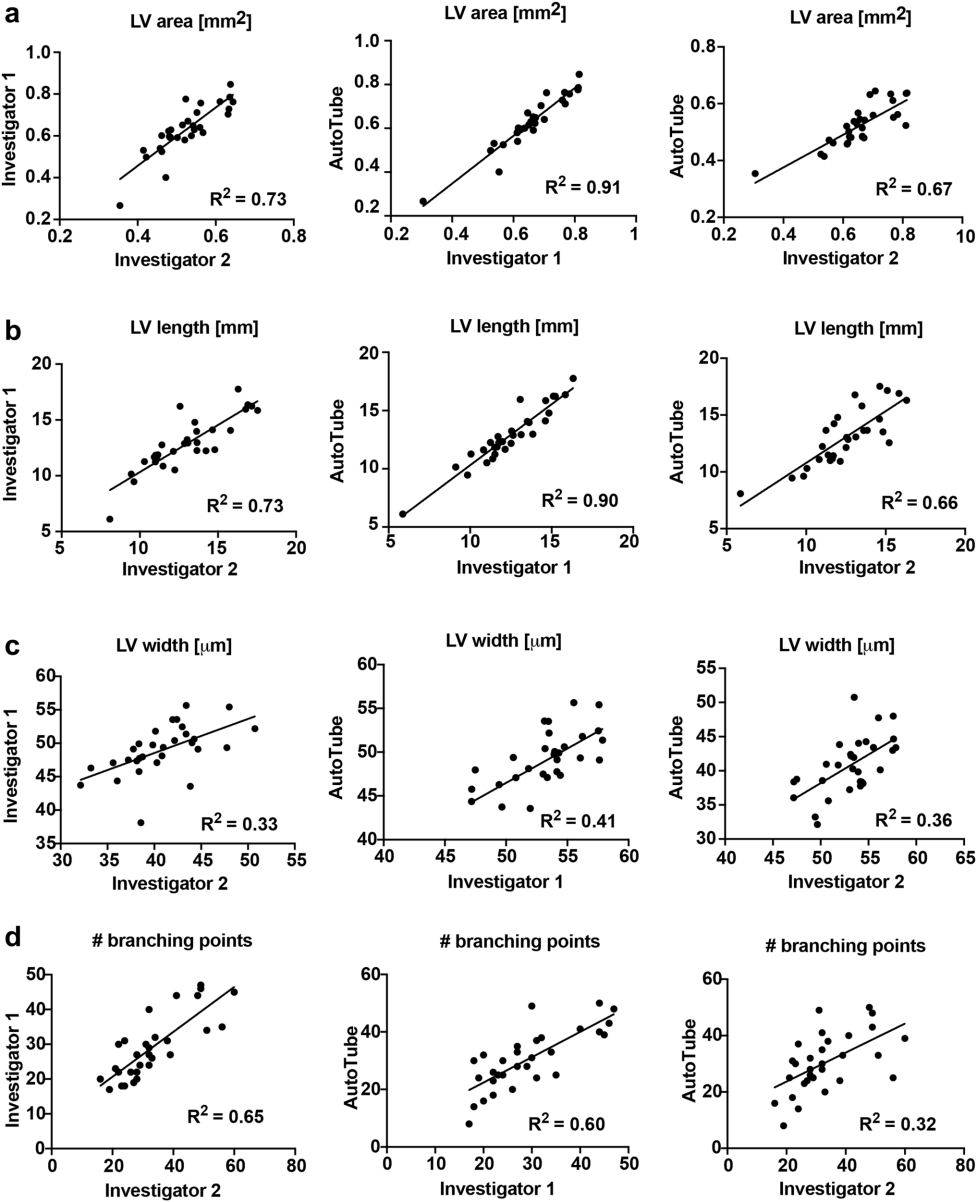
Analysis of the correlation between the two manual and the automated quantifications of all lymphatic vessel parameters analysed in Fig. 3. **a** lymphatic vessel area (LV area) **b** length (LV length) **c** width (LV width) of **d** # branching points. Pooled data of all images analysed (*n* = 30 images in total, from WT and IL7Rα^−/−^) are shown. The AutoTube settings used are described in Table 1

To better compare the datasets obtained by the manual and the automatic AutoTube-based quantification (Fig. 4), we next performed a correlation analysis. This analysis revealed that the *R*^2^ values achieved when comparing the manual datasets of investigator 1 and investigator 2 with each other were very similar to the *R*^2^ values obtained when comparing the datasets of each manual investigator with the dataset generated by the AutoTube software (Fig. 4). When cross-comparing all three analysis modes, high *R*^2^ values were consistently found for the lymphatic vessel area and length parameters (Fig. 4a, b). By contrast, more variability was observed for measurements of the lymphatic vessel width and of the number of branching points (Fig. 4c, d). Very comparable results were also obtained by manual and by automatic analyses of the lymphatic phenotype in the ear skin of IL7tg mice and in the diaphragm of ALCAM^−/−^ mice and their WT controls, respectively (Supplementary Fig. 3). For example, both the manual and the automated analysis detected a significant increase in the lymphatic vessel width of in IL7tg mice (Supplementary Fig. 3a, b) and an increase in the lymphatic vessel length and in the number of branching points in ALCAM^−/−^ mice (Supplementary Fig. 3c, d), in agreement with previously reported results [9, 42]. Thus, the software performed equally well as manual analysis in detecting and quantifying differences of the lymphatic network.

### AutoTube analyses blood vascular networks in a precise and reproducible fashion

**Fig. 5:**
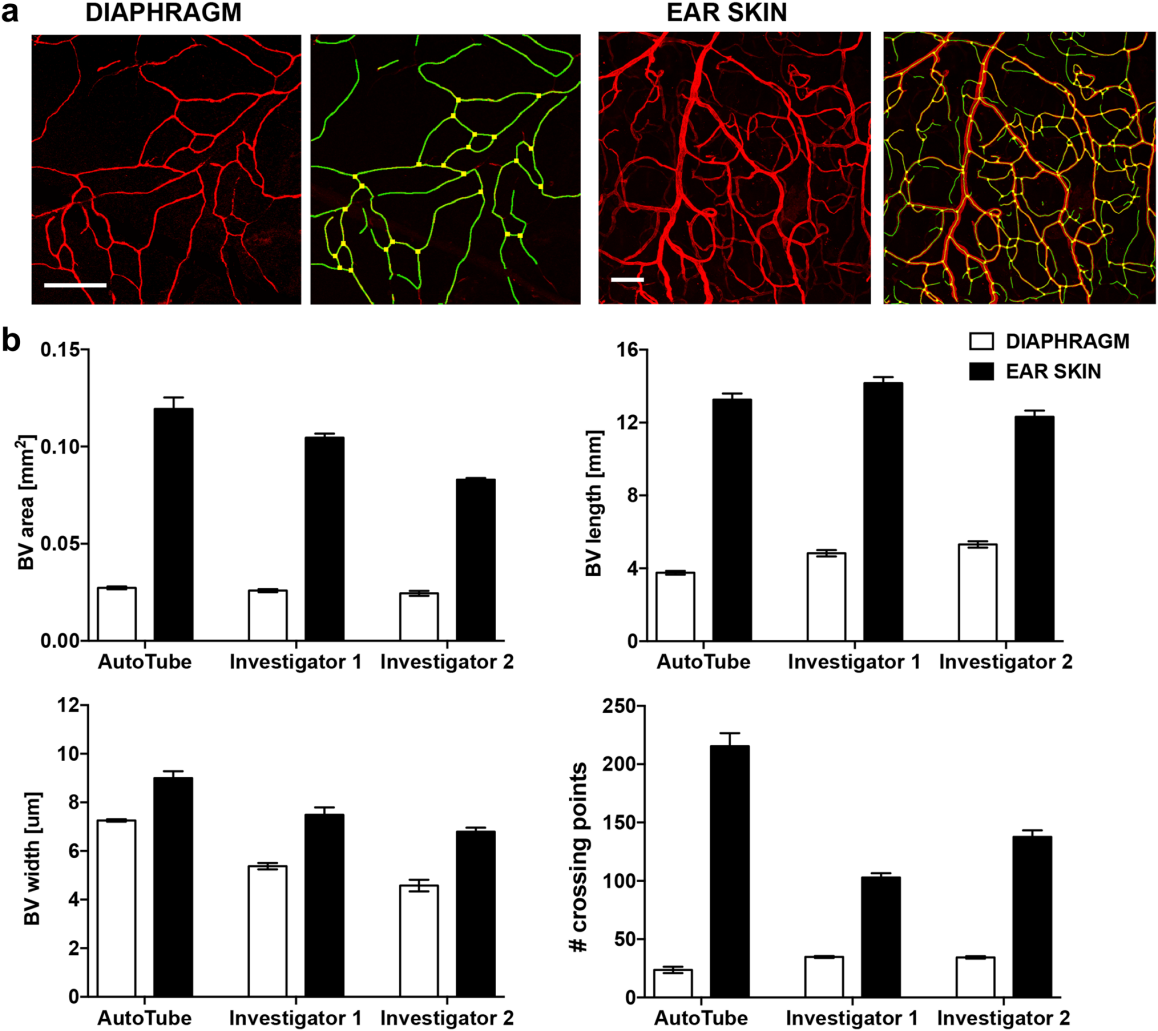
AutoTube-based and manual analysis of the MECA-32^+^ blood vasculature in tissue whole-mounts prepared from mouse diaphragms and ear skins. **a** Left: representative images of MECA-32 staining in the tissue indicated. Scale bar: 100 µm. Right: segmentation performed by the software. Detected blood vessels are shown in orange and blood vessel crossing points in yellow. **b** Automated and manual analysis detected similar changes in the vascular network in the diaphragm compared to ear skin. The data shown represent the mean ± SEM; statistical analysis was performed with Student’s *t*-test. *n* = 5–6 mice (2–3 pictures/mouse). The AutoTube settings used are described in Table 1

We next investigated whether AutoTube would also be applicable for morphometric analyses of blood vessels in tissue whole-mounts. As for the lymphatic vessel analysis, we performed a side-by-side comparison of datasets acquired manually by two different investigators or using the AutoTube software. Specifically, we analysed MECA-32^+^ blood vessels in the diaphragms of pups (P6) and in the ear skin of adult mice (both WT). As shown in Fig. 5a, the software was able to segment the acquired images from both tissues in an accurate fashion. A similar increase in the blood vessel area, length and width in the ear skin as compared to the diaphragm was observed in the two manual and in the automated analyses (Fig. 5b). Moreover, the correlation analysis performed revealed similar *R*^2^ values for the above-mentioned parameters across all comparisons (Supplementary Fig. 4a–c). While blood vessels in the diaphragm form a rather loose network and are preferentially present in one plane, they form a much more complex network the ear skin (Fig. 5a). In fact, the 2D projections of the 50–70-µm-thick confocal image stacks displayed many intersections of vessels crossing each other in different planes, making it impossible to distinguish between true and false-positive blood vessel branching points (Fig. 5a). As an alternative parameter of network complexity, we therefore performed a manual and AutoTube-based quantification of blood vessel crossing points. Interestingly, AutoTube detected significantly more, true-positive crossing points as compared to the two manual analysers (Fig. 5b, Supplementary Fig. 4d), likely because the latter often missed crossing points formed by faint vessels or crossing points that were in immediate proximity to each other (Supplementary Fig. 5a and data not shown). Taken together, AutoTube performed equally well to the manual analysis in detecting and quantifying differences in blood vessel morphology.

### Software-based analysis of an in vitro LEC tube formation endpoint experiment showed high correlation with manual analysis

**Fig. 6:**
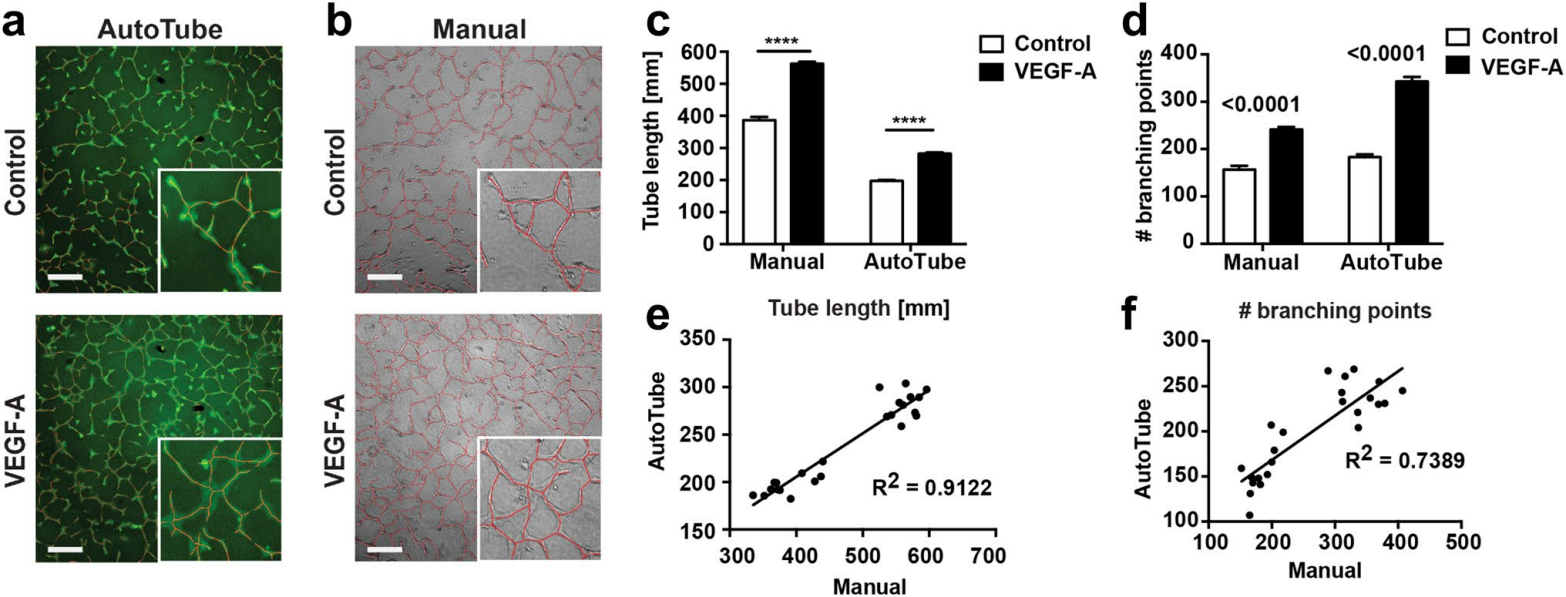
AutoTube-based and manual analysis of an in vitro LEC tube formation endpoint experiment. (a, b) Tube-like structures formed in vitro into a collagen matrix were stained at the end of the experiment with a fluorescent Cell Tracker Dye. Subsequently, bright-field and fluorescent two-dimensional images were acquired for manual and automated analysis, respectively. Representative images of control-treated or VEGF-induced LEC tube structures overlaid with corresponding skeleton images (red lines) generated by **a** the AutoTube software (fluorescence) and **b** manual analysis with Fiji (bright-field). Scale bar: 500 µm. **c** and **d** Total tube length and branching points per analysed picture quantified manually or automatically using AutoTube software. Results from one out of 3 similar independent experiments are shown. Data are shown as mean ± SEM. **e** and **f** Linear regression reveals high correlation between automated and manual analysis of area and branching points of tube-like structures (*n* = 20 pictures per condition). The AutoTube settings used are described in Table 1

Endothelial cell tube formation represents one of the most frequently used cell culture experiments to model (lymph)angiogenesis in vitro. For this reason, we investigated whether the AutoTube software would also be able to detect fluorescently labelled LEC tube-like structures within a collagen matrix in vitro and to compute common in vitro tube formation parameters. Indeed, AutoTube was able to detect fluorescently labeled tube-like networks formed in collagen at the time point of maximal tube formation (Fig. 6a, b). Moreover, in line with the manual analysis, the software also faithfully detected the VEGF-A-induced increase in overall tube length and in the number of branching points (Fig. 6c, d). For both parameters, the values computed by AutoTube correlated highly with the measurements derived from the manual analysis (Fig. 6e, f). Thus, the software provides an automated, reliable and objective way to analyse cellular tube formation experiments in vitro.

## Discussion

In this study, we describe the generation and validation of AutoTube, a new software for the automatic detection and analysis of tubes in vascular networks. To validate AutoTube, different datasets were analysed and compared to manual annotations performed by two independent investigators. Our results show that the automated analysis of lymphatic and blood vessel data by AutoTube faithfully reproduced the outcome of the manual analyses (Figs. 3, 5). Importantly, the automatic analysis reached the same scientific conclusions with very comparable measured values.

A major benefit of using automated analysis is that it is less labour-intensive compared to the manual analysis. On average, a 512 × 512 pixel image takes approximately 1 min when analysed by the software, while it can take up to 40 min when analysed manually. For a dataset with 24 images, the manual analysis thus takes up to 16 h, whereas AutoTube only needs to run for a maximum of 30 min. Another major advantage of AutoTube is that it bypasses the problem of inter-experimenter variability. When comparing the manual analyses performed by two different investigators, we found that the measured relative differences between vascular parameters were comparable. However, we also observed considerable inter-experimenter variability for some parameters, particularly for the lymphatic vessel area, the number of lymphatic branching points and the width. In the case of the vessel area, one reason for this discrepancy could be that the area is manually measured by subjectively thresholding an 8-bit fluorescent image. Another reason for the inter-experimenter variability could lie in the staining quality of the images. Often, images are not homogenously stained or illuminated so that some of the regions covered by vessels may appear darker. In such a case, the manual annotation is sensitive to what the annotator actually sees. In addition, the annotations may differ because of the number of images the annotator has been exposed to. A person who has previously annotated many images becomes a skilled annotator, who is capable of detecting vessels even in unevenly stained or noisy regions. To identify the best suited parameters for an AutoTube-based analysis of a given dataset (Table 1), different parameter combinations are ideally compared with an initial manual analysis performed on the same dataset. Consequently, these initial AutoTube-based analysis results will be skewed (“trained”) towards the results obtained by the initial manual analyser, implying a certain degree of subjectivity. However, once the best parameters have been defined and set, the software will analyse all subsequent datasets in the same manner, thereby allowing for a more objective comparison of different experiments/datasets.

Apart from generating data that support the same scientific conclusion, the AutoTube software also was rather accurate in generating absolute values that were similar to those obtained by manual analysis. As shown in Fig. 4 and Supplementary Fig. 4, datasets generated by AutoTube highly correlated with datasets generated by manual analyses, particularly for parameters like the vessel area and vessel length. In these cases, the obtained *R*^2^ values were very similar to those obtained when comparing the two manual datasets with each other. This cross-comparison confirms the ability of AutoTube to perform the morphometric image analyses with a similar accuracy as the manual investigators.

Interestingly, in the case of blood and lymphatic vessel width, the cross-comparison analysis revealed that all *R*^2^ values were rather low. The fact that these measurements poorly correlated across all comparisons indicates that this is an intrinsic problem of the vessel width parameter. In fact, the LV or blood vessel width is calculated by dividing two variables, namely the vessel area by the vessel length. The low correlation between the lymphatic and blood vessel width seen across all the annotations could therefore be related to the propagation of errors coming from the individual area and length measurements. Likewise, we observed that there occasionally was a poor correlation between the different manual and the automatic measurements of lymphatic vessel branching points (Fig. 4d), or of blood vessel crossing points (Fig. 5b, Supplementary Fig. 4d). In the case of blood vessels AutoTube turned out to perform better than the manual analysers in detecting crossing points in a given whole-mount image. However, in the case of lymphatic vessels, AutoTube occasionally detected false-positive branch points: This problem typically occurs when the staining quality of an image is poor and/or when vessel branches in the image overlap. For example, when the staining of lymphatic vessels is heterogeneous, AutoTube occasionally detects false branches and branching points within single vessel segments (Supplementary Fig. 5b). Moreover, given that AutoTube works with image projections of confocal 3D image stacks and thus performs a strictly 2D analysis of the vascular network, the software considers two lymphatic vessels that spatially overlap or cross as a branching point (Supplementary Fig. 5c). By contrast, a manual annotator is to a certain extent capable of extracting 3D information from the 2D image stack projection and consequently may be able to discriminate between a true and a false lymphatic vessel branching point. As a potential solution to these problems, we have included a “brush” tool into the software, which allows to correct for small mistakes: i.e. inaccurately detected vessels or branching points in images with high background can be deleted, or patchy and heterogeneous stainings can be filled to allow for an accurate detection by the program. However, this type of manual intervention may again add a level of subjectivity to the otherwise completely automated analysis.

In the vascular biology field, few automated tools are currently available and broadly used to analyse complex *ex vivo* and in vitro vascular network data. The commercial software IMARIS (Bitplane) possesses a feature named “Surface” that allows to perform whole-mount and tube formation analyses. However, this tool copes poorly with heterogeneous, patchy stainings and offers a very limited possibility for manual correction (data not shown). Another software available for quantitative analysis of angiogenesis is “AngioTool”, which was developed by the National Cancer Institute [43]. However, similar to IMARIS, AngioTool does not offer the possibility to correct for high background in pictures or to delete erroneous detections. Furthermore, it has been specifically refined for the analysis of blood vascular networks and not for lymphatic vessels or for in vitro assays. By contrast, AutoTube is highly versatile as it may be used for the analysis of blood vascular and lymphatic networks in different tissues and for in vitro assays. Therefore, AutoTube represents a fast, automated and highly versatile software for objective and reproducible analysis of vessel data.

## Electronic supplementary material

Below is the link to the electronic supplementary material.

Supplementary material 1 (PDF 7841 KB)

Supplementary material 2 (PDF 16011 KB)

## Footnotes

1 BM3D v2.0 was acquired from http://www.cs.tut.fi/~foi/GCF-BM3D/.
