# Supplementary material for: AutoTube: a novel software for the automated morphometric analysis of vascular networks in tissues
Source: Angiogenesis. 2018 Oct 28;22(2):223–36. doi: 10.1007/s10456-018-9652-3 (PMC6475513; doi:10.1007/s10456-018-9652-3)
Supplement: Supplementary file 1 — Supplementary material 1 (PDF 7841 KB) [file 10456_2018_9652_MOESM1_ESM.pdf]

AutoTube: a novel software for the automated morphometric analysis of vascular networks in tissues

Javier Montoya Zegarra[2¶], *Erica Russo*[1¶], *Peter Runge*[1¶], *Maria Jadhav*[1], *Ann-Helen Willrodt*[1], *Szymon Stoma*[2], *Simon F. Nørrelykke*[2], *Michael Detmar*[1] *and Cornelia Halin*[1]

[1] Institute of Pharmaceutical Sciences, ETH Zürich, Vladimir-Prelog-Weg 1-5/10, 8093 Zürich, Switzerland

[2] Scientific Center for Optical and Electron Microscopy (ScopeM), ETH Zürich, Wolfgang-Pauli-Str. 14, 8093 Zürich, Switzerland

[¶] these authors contributed equally to this work

Corresponding author:
Cornelia Halin, Ph.D.
e-mail: cornelia.halin@pharma.ethz.ch
phone: +41 44 633 29 62

AutoTube Manual

This file contains the licence agreement, installation and user guideline for AutoTube. AutoTube quantifies vascular parameters such as the vessel area, width, skeleton length, and the number of branching points from 2D images of vascular networks in tissues or in *in vitro* assays. AutoTube is freely available, comprises a simple Graphical User Interface (GUI), and helps to perform otherwise highly time-consuming image analyses in a rapid, automated objective, and reproducible manner.

The software is provided as a source code and is available on GitHub under https://github.com/autotubularity/autotube. It has been tested on three different operating systems: macOS (10.12 Sierra and 10.13 High Sierra), Windows 8 and 10, and Ubuntu 16.04. We suggest to run the application on computers with at least 8GB of RAM Memory.

Since the source code of AutoTube is freely available, this offers the possibility for further adaptation or extension of the software according to the specific research needs. An exact copy of the software at the time of the paper acceptance has also been deposited under the following link: https://tinyurl.com/y9sopo94. It is intended for archiving purposes.

Potential users of AutoTube can run the software online prior to using it locally on their own machines. To do so, we have created a password-protected Virtual Machine (VM). To obtain access to the VM, interested users are encouraged to contact the corresponding author by e-mail.

Table of Contents:

1) Licence Agreement

2) Source Code

3) Graphical User Interface (GUI)

4) Source Code Functions

Copyright 2018, Pharmaceutical Immunology Group of the Institute of Pharmaceutical Sciences (IPW) of ETH Zurich (ETHZ): http://www.pharmaceutical-immunology.ethz.ch

1. Licence Agreement

AutoTube is an open source software. If you use all or any portion of this software, we ask that you quote the original paper (Montoya, Russo, Runge et al., Angiogenesis 2018) in which AutoTube was described.

2. Source Code

The source code of AutoTube can be accessed from the following link: https://github.com/autotubularity/autotube

To run AutoTube from the source code, you have to download the autotube_source.zip file from the link and store it locally. After uncompressing the file, three directories will be extracted:

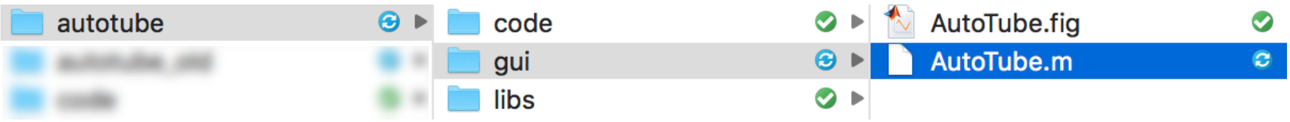

The *code* directory contains the image processing functions to analyse the images. The *gui* directory contains the AutoTube graphical interface. Finally, the *libs* directory contains third-party libraries used by AutoTube. To run AutoTube directly from Matlab, double-click on the *AutoTube.m* file contained inside the *gui* directory. Then follow the next steps:

Step 1: Once Matlab is opened, click on the *Run* green button on the Editor Toolbox (as shown in the red rectangle in the Figure below):

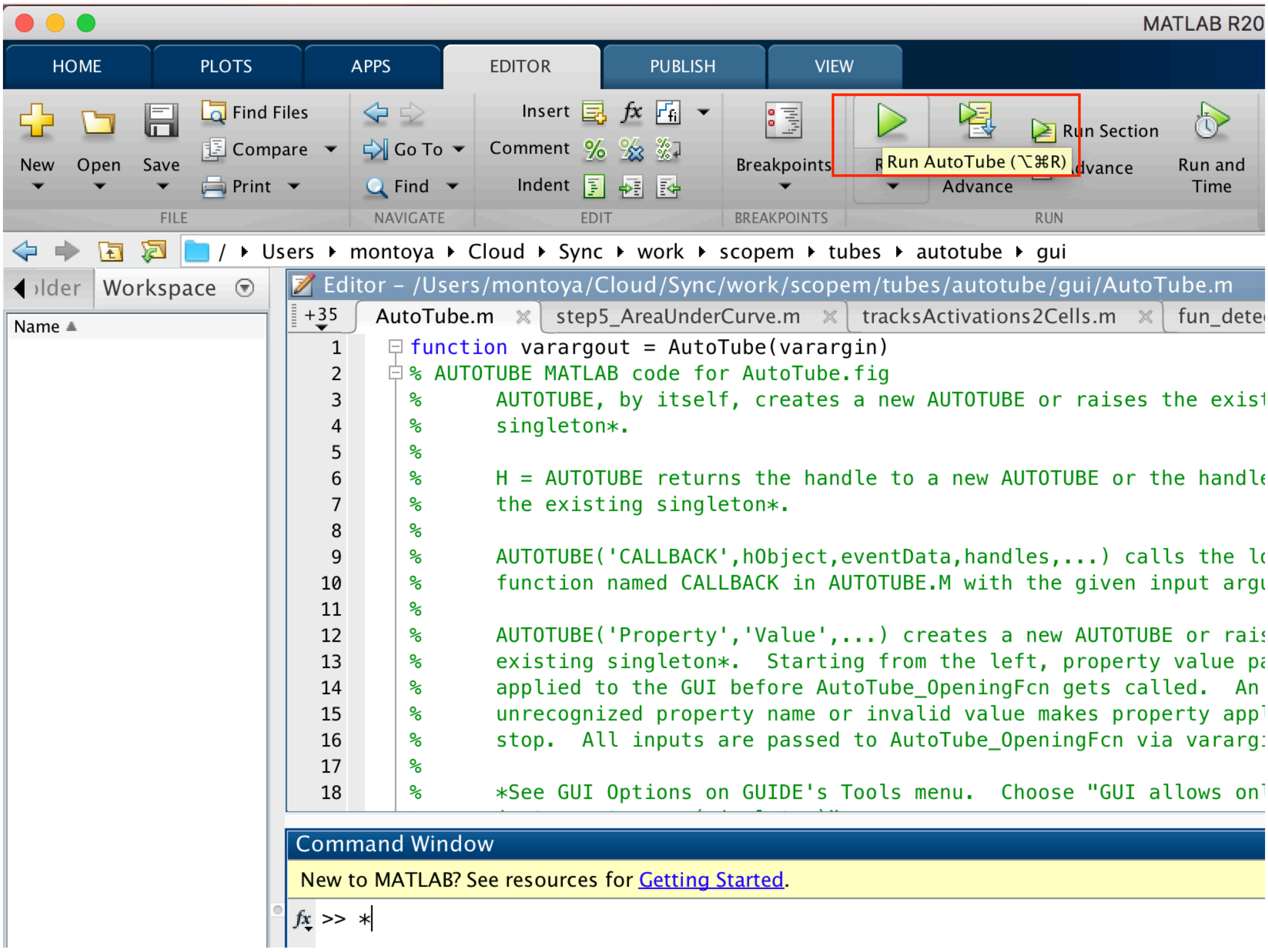

Step 2: After clicking on the *Run* button, the AutoTube GUI is opened and is ready to use. You should get the same Figure below:

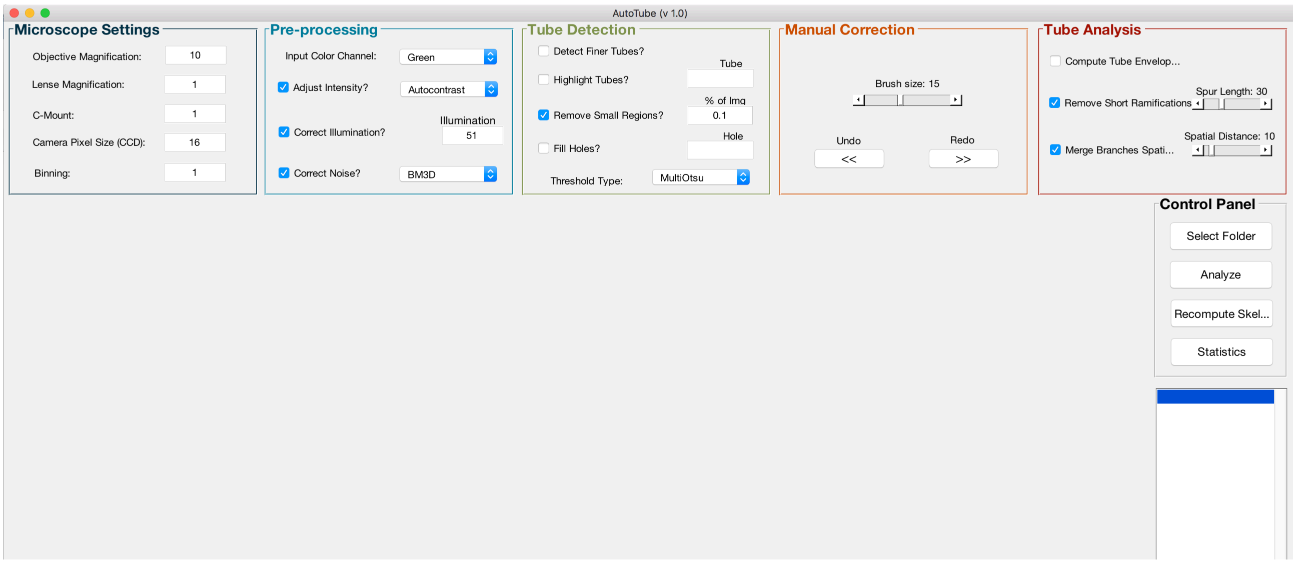

Another possibility of running AutoTube from Matlab is to use the *guide* command from the Matlab Console. To do so, follow the next steps:

Step 3: Type *guide AutoTube.fig* in the Matlab Command Window as shown in the Figure below:

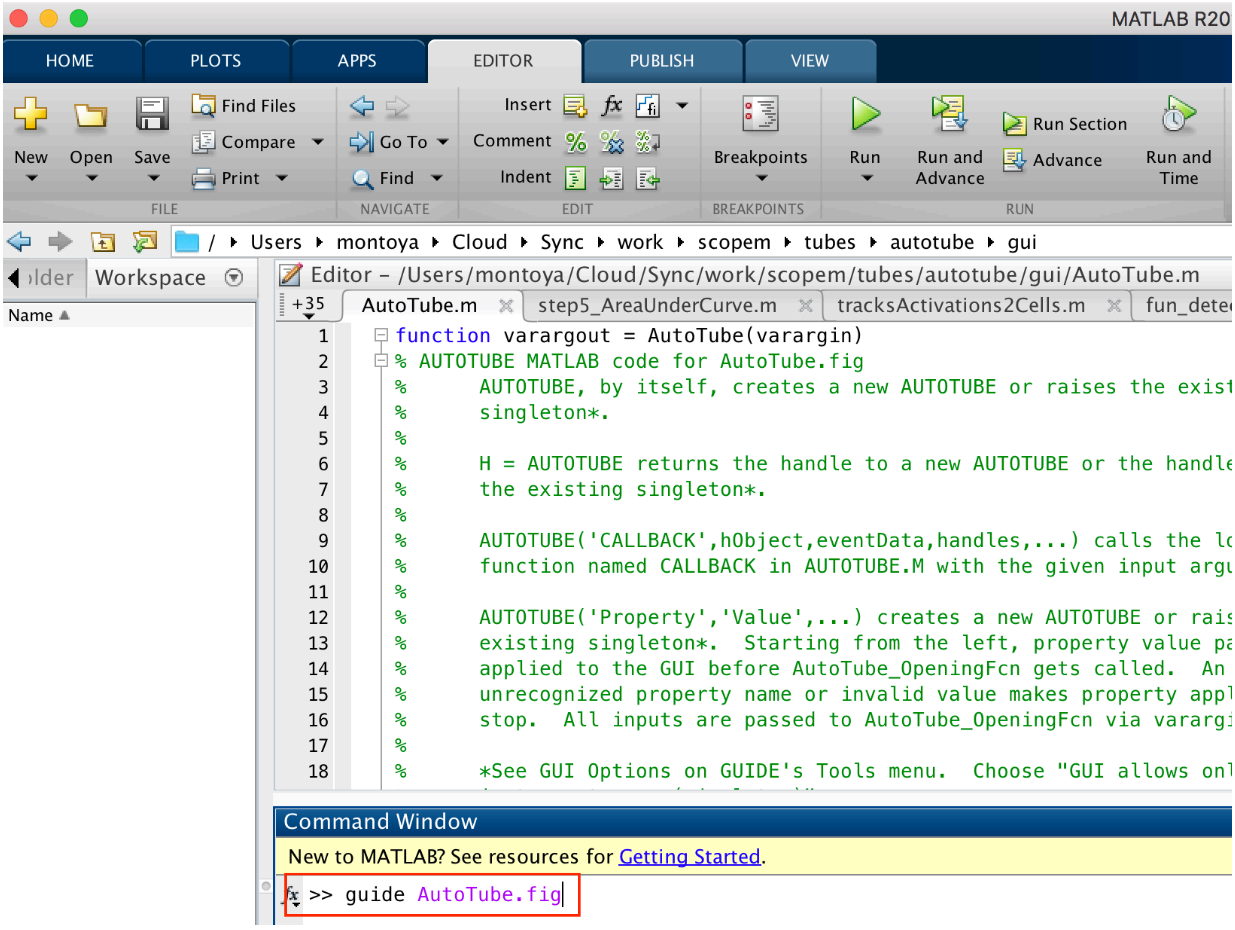

Step 4: The AutoTube.fig GUI is opened. Click next into the *Run* green button:

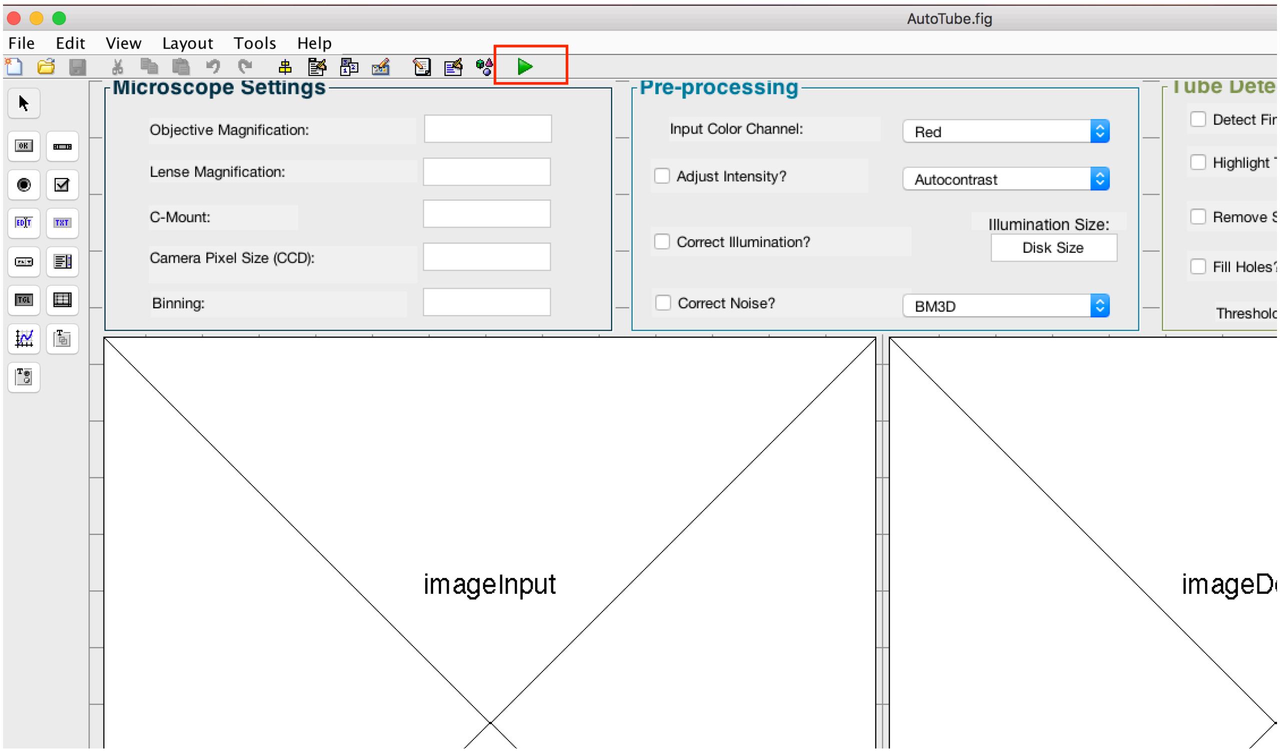

Step 5: The AutoTube GUI is opened and is ready to use.

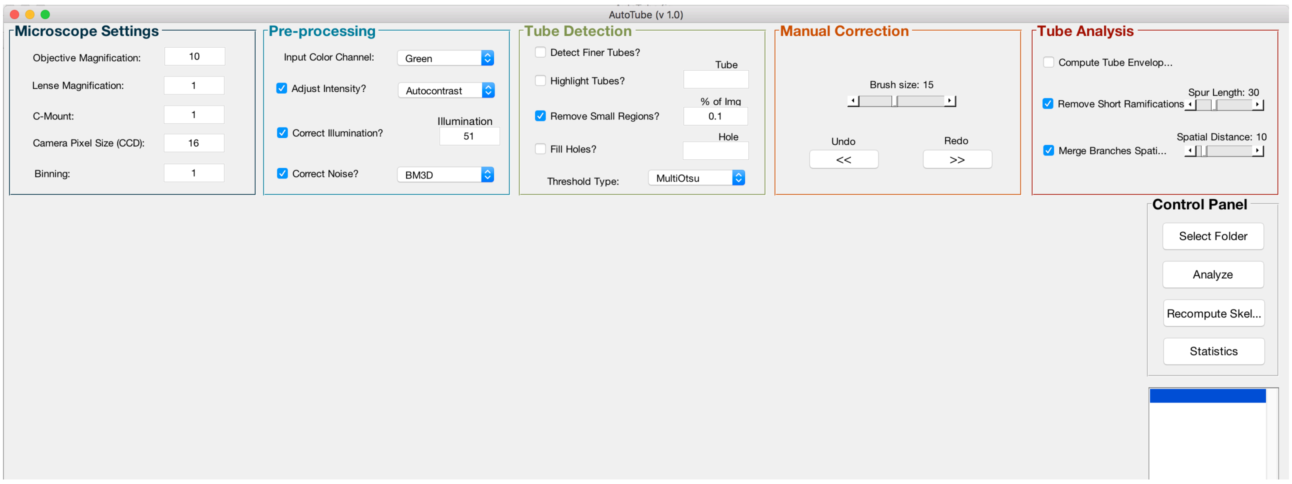

Step 6: To validate that AutoTube is running smoothly, you can process one test image. To do so, download the test image named ***test_image.tif*** from the following link: https://tinyurl.com/ycnbmmxh. Store the image locally to your hard drive. Note that also the automatic results from AutoTube are going to be stored in this directory. In the example below, the .tif image was downloaded into the MATLAB directory under Documents.

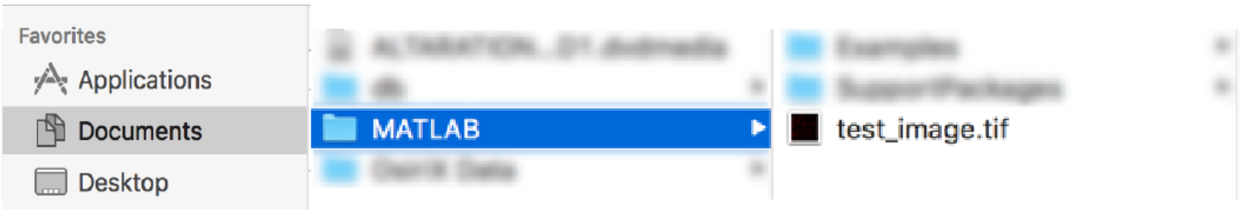

Step 7: In the AutoTube GUI click "select folder" (No. 1 in the Figure below) to choose the directory containing the image, in our example /Documents/MATLAB". Next, adjust the "input color channel" to *red* (No. 2 in Figure) and set the "remove small regions" value to *0.2* (No. 3 in Figure). To run the analysis, click on the button "Analyze" (No. 4 in the Figure below).

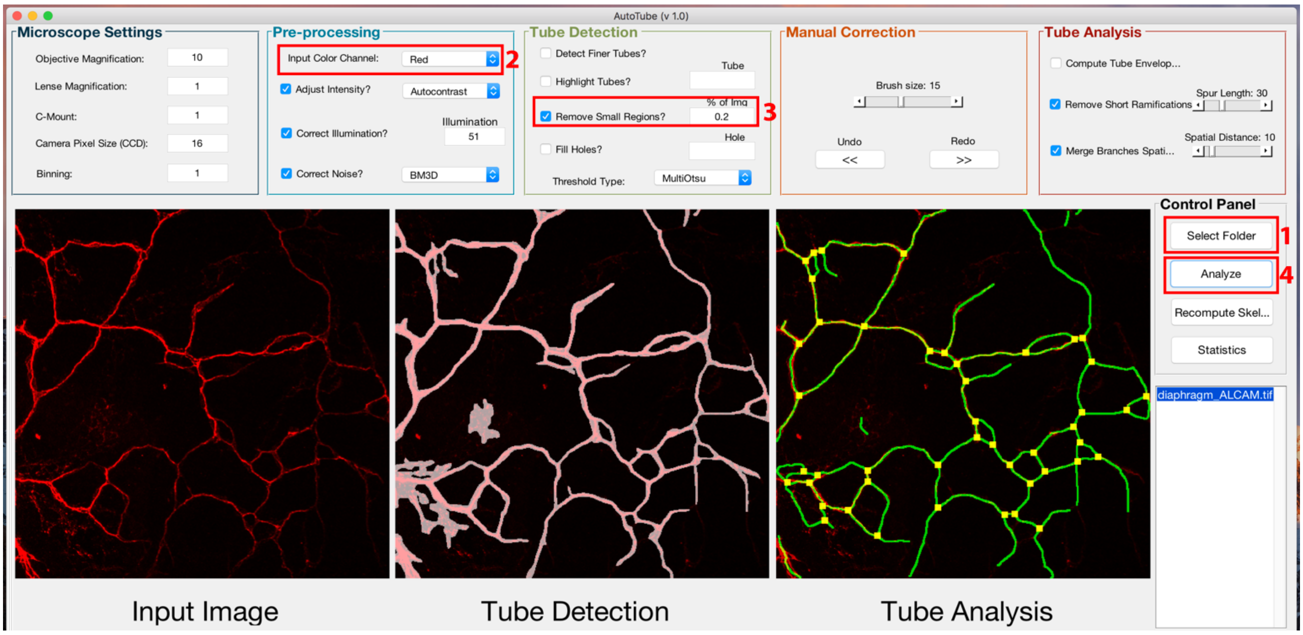

Step 8: Once the analysis is finished, you will find a new directory named *"output"* at the same location, where the test image was downloaded. You will find additional subdirectories containing the results from the image processing steps conducted by AutoTube.

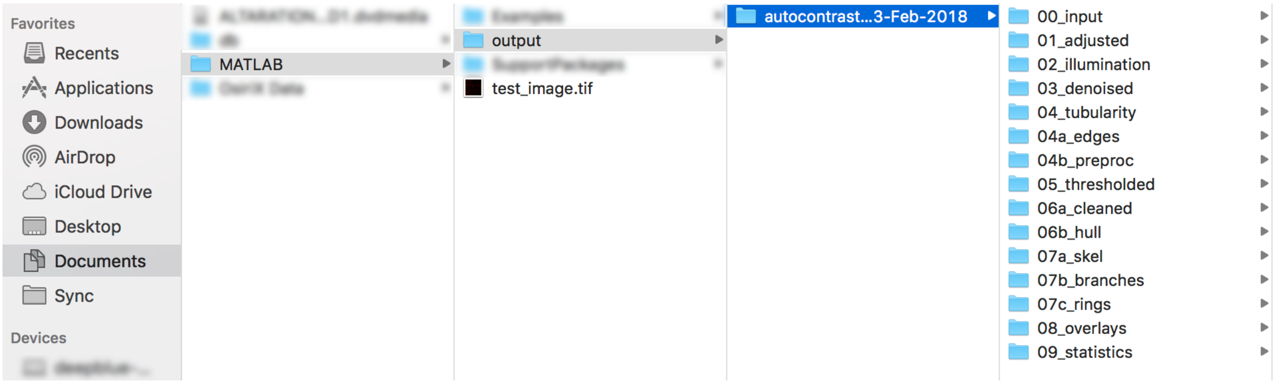

Important note:

The BM3D version included in the *source code* of AutoTube, when running on Linux or Mac OS operating systems (*Beta version, obtained directly from the authors and to be soon released under http://www.cs.tut.fi/~foi/GCF-BM3D/*), is different from the BM3D v2.0 version used when running on Windows (*v2.0, released on January, the 30th of 2014 under*

http://www.cs.tut.fi/~foi/GCF-BM3D/). This is due to the fact that the current official BM3D v2.0 is not compatible with the newest versions of Mac OS in combination with Matlab versions > R2017a. To solve this issue, the authors of BM3D are planning to officially release soon the modified *Beta* version, which is already included in the *source code* of AutoTube.

Please note that our validation experiments confirmed that the use of the different BM3D versions only has a minute impact on the overall analysis results. Specifically, we analysed an entire vascular data set (belonging to Figure 3 and Supplemental Figure 2 of the AutoTube manuscript) with AutoTube that either employed the Windows- or the MacOS-based BM3D version. In this comparison both BM3D analysis modes delivered highly similar results, in terms of absolute values measured, standard deviations and biologic interpretation (see **Figure 1**, below). Thus, no major differences in the results are expected when using either of the two AutoTube-embedded BM3D analysis modes.

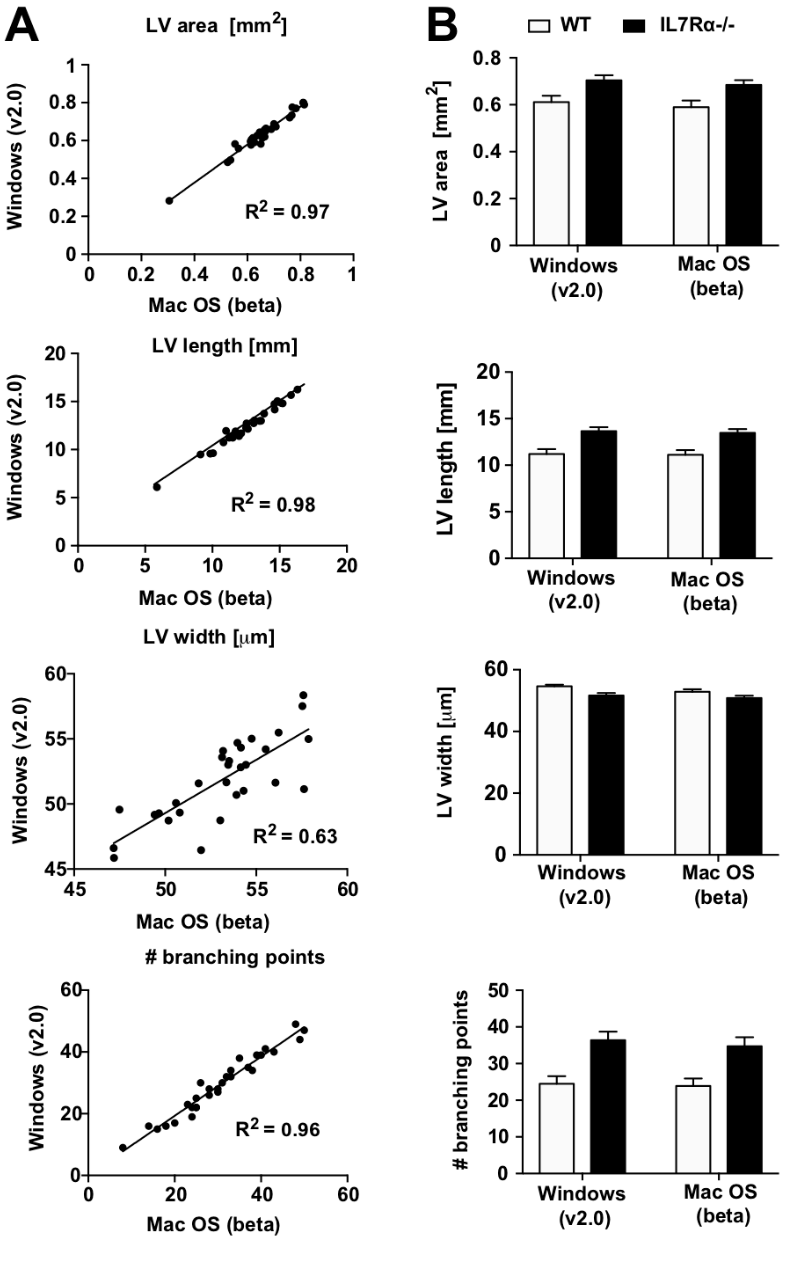

Figure 1: **Comparison of the impact Windows- (version 2.0) or Mac OS-based (beta version) BM3D-denoising on the analysis of the lymphatic vasculature in WT or IL-7Rα$^{-/-}$ murine ear skin.** AutoTube was run on the same image dataset using either the Windows- or Mac OS-based BM3D-denoising mode. The dataset used for the analysis is the same as for Figure 3 and Supplemental Figure 2 of the AutoTube paper and can be downloaded from *https://doi.org/10.3929/ethz-b-000262426*. Parameters analysed include the area covered by lymphatic vessels (LV area), the absolute length of the skeleton (LV length), the average vessel width (LV width) and the number of branching points (# branching points) per picture. Correlation analyses are shown in (**A**) and standard column graphs in (**B**).

3. Graphical User Interface (GUI)

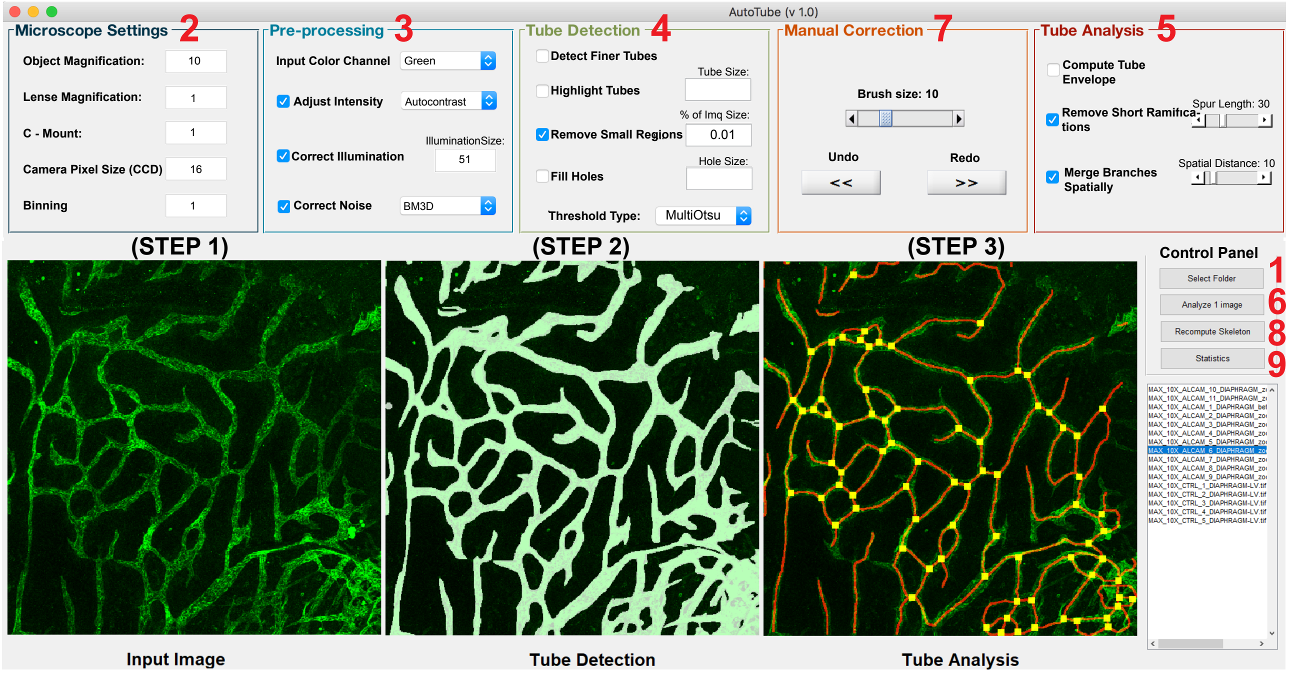

***Figure 2**: Screenshot of the Graphical User Interface (GUI) of AutoTube. The individual steps (numbers in **RED**) are explained in detail in the text below.*

In this manual, we describe the necessary steps that the User needs to follow to analyse a set of tube images. In addition, the different parameters of the GUI are explained and suggested values are proposed. We tried to make the AutoTube GUI intuitive and easy to use. In the following, the most important steps for the correct usage of the software are explained. Note that each of the steps are associated with a number in red in **Figure 2**.

Step 1: Click on the "Select Folder" button to locate the directory containing the (*.TIF, *.JPEG, *.PNG) files of your experiments. You should select the top directory containing the images. After that, click "Enter". You'll next see that the list of image filenames gets displayed.

Step 2: Microscope Setting parameters. These parameters are related to the microscope and are used to convert distances from pixels to μm.

There are five main parameters:

- Object Magnification: this is the power of the microscope objective. Example values are: 10x, 20x, and 40x.
- Lens Magnification: extra magnification of e.g. 1.25x, 1.6x, or 2x. Leave it at 1 if your microscope does not have any extra lens magnification.
- C-Mount: is usually set to 1. Other values, e.g. 0.45x, may be used if a camera adapter with that power of magnification is in place.
- Camera Pixel Size (CCD): linear size in μm of a physical pixel (assumed to be square) in the CCD chip of the camera used. Typical values are in the range 0.5 - 16 μm.

- Binning: combines pixels in the camera to one single pixel in the acquired image - typically used under low light conditions. The common options are 1 (1X1), 2 (2X2), or 4 (4x4). Set this value to 1 as default and it represents that each pixel in the camera is mapped to its own pixel in the output image.

The final image pixel size is given by the following equation:

pixel size = camera pixel size * binning / (obj. mag. * lens mag. * c-mount)

Step 3: Pre-processing parameters for the input images. These parameters are responsible for reducing detrimental effects from image acquisition, such as poor contrast, uneven illumination or noise (For more details, please read the *Image Pre-processing* subsection in the *Materials and Methods* Section of the paper). There are four main parameters to be tuned:

- Input Colour Channel: the colour channel over which the analysis is going to be performed.
- Adjust Intensity: choose whether an intensity adjustment correction shall be performed. You can choose among three different options: auto-contrast operation, global histogram equalization, and adaptive histogram equalization. The auto-contrast and histogram equalisation options are useful when the contrast across the different image regions is homogeneous, otherwise, use adaptive histogram equalization.
- Correct Illumination: choose whether the illumination should be normalized and set the approximate diameter size (in pixels) associated with the circular uneven illumination effect. Note that this parameter is related to the microscope lenses diameter in pixels. The lenses diameter size that we considered for both lymphatic (LV) and blood vessels (BV) was set to 51, and for the acquisition settings of our images, a value in the range 40-60 pixels.
- Correct Noise: choose whether noise shall be removed from the input images. Options include: BM3D or Wiener Filtering. BM3D is a more robust de-noising method that should be preferred and is the default option for image de-noising. The Wiener filter is a less computationally heavy approach, preferred when results should be obtained faster.

Step 4: Tube-Detection parameters. They are responsible for the actual partition of the images into foreground (vessels) and background regions. For more details, refer to the *Tube Detection* subsection in the *Materials and Methods* section of the paper. Five main parameters can be tuned here:

- Detect Finer Tubes: when selected, finer tubes are detected by looking for image regions containing edges.
- Highlight Tubes: when selected, the Frangi Vesselness Filter is applied. The User also has to give as input an estimate in pixel values of the width of the tubes. This option is

especially useful when the quality of the staining is low and also when the images exhibit some artefacts that do not belong to the tubes. We saw empirically that for blood vessels, the values of 3, 5, or 7 obtained good results, while for lymphatic vessels, we used values of 11, 13, or 15.

- Remove Small Regions: after thresholding, regions whose area is below a given percentage of the total image size, are removed. By default, all regions smaller than 1% of the total image size are removed.
- Fill Holes: after thresholding, it is possible to fill holes/gaps in the detected vessels. The User has to define the area in pixels of the holes to be filled.
- Threshold Type: The User can select different Thresholding techniques including, Otsu/Multi-Otsu, Kittler, and Adaptive (CLAHE). The Otsu Thresholding technique is the most conservative Thresholding option. This option is recommended when the images are especially clean and staining is strong. Multi-Otsu is an extension of the Otsu Method, in which additional tubes are detected by considering more pixel classes. In our analysis, we fixed the number of pixel classes/modes to 3, one for the background pixels and the other two for the vessels. Especially when the staining is weak, the third class covers vessel regions that are otherwise not detected. Multi-Otsu is the default option. The Kittler method is sensitive to local noise, however it is able to detect tubes even in weak stained images. Finally, the Adaptive method achieves especially good performance when the images exhibit uneven illumination factors in different regions. *For more details and references, please read the Materials & Methods section of the AutoTube manuscript.*

Step 5: Tube-Analysis parameters. They are used when computing the morphometric properties of the detected vessels. In specific, they are useful for post-processing the detected tubes.

- Compute Tube Envelope: this option computes the convex-hull over the detected tubes (tube-region envelope) and returns the area of the detected tubes over the area of the envelope. This value is subsequently stored in the output statistical file.
- Remove Short Ramifications: this option removes the ramifications of the skeleton that are shorter than a given pixel value. The User selects the minimum-length for the valid ramifications. For BVs, we found that a good initial value is 30pix and for LV we set this parameter to 15pix, when both images are acquired using a 10x objective.
- Merge Branches Spatially: when the quality of the stainings is low, some image regions appear pixelated. Therefore, the software occasionally detects too many branching points in close proximity. Branching points that are very close together are typically false-positives and therefore can be merged. The User defines the radius of the circular regions over which the branching points are averaged. We found that for BV this parameter can be set up to 15pix (~ 25 μm), while for LV we used a parameter value of 10pix (~ 16.7 μm).

For more details, please read the *Tube Analysis* subsection in the *Materials and Methods* Section of the paper.

Step 6: Analysis. Once the parameters are configured and the User clicks on the "Analyse Image" button, the selected images are processed and the morphometric properties are computed. Note that the label of the button gets automatically updated based on the number of images selected, i.e if only one image is selected, then the label of the button is "Analyse 1 Image", however there is no limit on the total number of images selected for the analysis. Once an image is analysed, then the next image gets automatically processed until all selected images are analysed. On the other hand, a total number of 9 output directories are created on disk to store the output after applying each image processing step. Note that storing the images after each processing step helps the User to have a better understanding of the effect of the parameter values. The User can next adapt the parameters accordingly. Examples of directories include the "01_adjusted" directory, where the images after intensity-adjustment are stored. In the directory "08_overlays", the final skeleton and branch points are stored for each image. Finally, in the directory "09_statistics", an excel file is created containing the morphometric properties for each processed image.

Step 7: Manual Correction. It is possible that after the automatic analysis of the images some *false-positive* or *false-negatives* tubes might need to be corrected. To do so, the User can paint/add circular regions corresponding to "missing" tubes by left-clicking with the mouse on the "Tube Detection" Image in Tube Detection panel. This will add for each click a circular spot. Also, to remove false-positive tubes, the User needs to right-click with the mouse, the regions that should be removed, on the "Tube Detection" Image in Tube Detection panel. Every time this is executed, the User can adapt the size of the Brush, by changing the parameter value in the Brush-slider.

Step 8: Recompute Skeleton. After having manually corrected the detected tubes, the morphometric tube properties (e.g., skeleton, branch points) need to be recomputed for the new tubes. To do so, the User needs to click on the "Recompute Skeleton" button. Once the processing is finished, the new skeleton and branching-points are displayed in the "Tube Analysis" panel. If needed, the User can modify again the detected tubes. Note that for each single image that needs to be updated, this step needs to be repeated.

Step 9: Statistics. Once the User is satisfied with the detected tubes, the "Statistics" button is clicked to generate a csv file containing the statistics of the morphometric tube properties. The excel file can be found in the "09_statistics" directory that is found in the "output" directory, at the same location, where the images are stored on disk. The name of the file is stats_summary_skelcut{digit1}_branchcut{digit2}.csv The variable *digit1* corresponds to the value used for defining the minimum-length of valid ramifications (see Step 4). The variable *digit2* corresponds to the value of the radius set to average the branching points (see Step 4).

Note that the csv file will contain the extracted geometrical properties in both *pixel* and *µm* units. For that purposes, the column names in the csv file will be followed respectively by "(*pix*)" or "(*µm*)" in their titles to denote the used units.

4. Source Code Functions

In this Section, we present the core image processing functionalities of AutoTube. They involve: (i) intensity adjustment, (ii) correction of uneven illumination, (iii) image denoising, (iv) tube detection, and (v) tube analysis. For each of these functionalities, a description on the header of the function is included which contains the input and output variables of the given function together with a sentence explaining the goal of the given function.

```
function im_adj = doAdjust(im, opts)
% doAdjust: this function adjust the intensity of a given input image. By
% default an "adaptive histogram equalization" transform is applied.
%
% inputs:
%       im: a 2D gray-level image.
%       opts: a matlab structure containing the type of intensity
%           -> opts.adjustment (string) adjustment to be applied,
'autocontrast', 'global', or 'adaptive' (default)
%
% output:
%       im_adj: intensity-adjusted image.

    if ~isfield(opts, 'adjustment')
       opts.adjustment = 'autocontrast';
    end

    opts.adjustment = lower(opts.adjustment);

    if isempty(strfind(opts.adjustment, 'autocontrast')) == 0
        im_adj = imadjust(im);
    elseif isempty(strfind(opts.adjustment, 'global')) == 0
        im_adj = histeq(im);
    elseif isempty(strfind(opts.adjustment, 'adaptive')) == 0
        im_adj = adapthisteq(im);
    else
        im_adj = adapthisteq(im);
    end
end


function im_illu = doIlluCorrection(im, opts)
% doIlluCorrection: this function normalizes illumination from a gray-level
image
% using the top-hat transform.
%
% inputs:
%       im: a 2D gray-level image.
%       opts: a matlab structure containing the parameters for the
%             illumination correction. The default size of the circular
%             kernel is of 51.
% output:
%       im_illu: 2D illuminated-normalized image.

    if ~isfield(opts, 'illumination')
        opts.illumination = [];
    end

    if ~isfield(opts.illumination,'seSzeIllu')
```

```
        se = strel('disk', 51);
    else
        se = strel('disk', opts.illumination.seSzeIllu);
    end

    op  = imopen(im, se);
    im_illu = im - op;
end

function im_den = doDenoising(im, opts)
% doDenoising: this function denoises an input image, i.e. reduces noise.
% By default a BM3D-based denoising is applied.
%
% inputs:
%       im: a 2D gray-level image.
%       opts: a matlab structure containing the type of denoising to be
%               applied.
%       -> opts.denoising.type: (string) type of denoising to be applied: 'BM3D'
or 'wiener' filter.
%
% output:
%       im_den: denoised image.

    if ~isfield(opts, 'denoising')
       opts.denoising= [];
    end

    if ~isfield(opts.denoising, 'type')
       opts.denoising.type = 'BM3D';
    end

    if ~isfield(opts.denoising, 'bm3d')
       opts.denoising.bm3d = '';
    end

    if ~isfield(opts.denoising.bm3d, 'sigma')
       opts.denoising.bm3d.sigma = 100;
    end

    if strcmpi(opts.denoising.type, 'BM3D')
        im = im2double(im);
        if ispc
            [~, im_den] = BM3D_win(1, im, opts.denoising.bm3d.sigma);
        elseif ismac || islinux
            [im_den, ~] = BM3D_mac_linux(im, opts.denoising.bm3d.sigma/255.0,
'np');
        else
            error('Operating System not supported!');
        end
    elseif strcmpi(opts.denoising.type, 'wiener')
        [im_den,~] = wiener2(im, [5 5]);
    else
        error('Unkown denoising method: %s\n', opts.type);
    end
end
```

```
function imFrangi = doFrangi(im, opts)
% doFrangi: this function applies the Frangi filter on a given input image.
%
% inputs:
%       im: a 2D gray-level image.
%       opts: a matlab structure containing the parameters for the frangi
%               filter.
%
% output:
%       imFrangi: frangi-vesselness response.
%
% references: for more details on the method, refer to:
%             Frangi et al. Multiscale Vessel Enhancement Filtering

    if ~isfield(opts, 'tubularity')
        opts.tubularity = [ ];
    end

    if ~isfield(opts.tubularity, 'beta1')
        opts.tubularity.beta1 = 0.5;
    end

    if ~isfield(opts.tubularity, 'beta2')
        opts.tubularity.beta2 = 15;
    end

    if ~isfield(opts.tubularity, 'blackwhite')
        opts.tubularity.blackwhite = false;
    end

    if ~isfield(opts.tubularity, 'sigmaInit')
        opts.tubularity.sigmaInit = 1;
    end

    if ~isfield(opts.tubularity, 'sigmaRatio')
        opts.tubularity.sigmaRatio = 2; %sqrt(2);
    end

    if ~isfield(opts.tubularity, 'sigmaEnd')
        opts.tubularity.sigmaEnd = 5;
    end

    sigmas     =
opts.tubularity.sigmaInit:opts.tubularity.sigmaRatio:opts.tubularity.sigmaEnd;
    beta       = 2*(opts.tubularity.beta1)^2;
    correction = 2*(opts.tubularity.beta2)^2;

    filtImArr = zeros([size(im) numel(sigmas)]);

    for ii=1:numel(sigmas)

        [ixx, ixy, iyy] = do_hessian(im, sigmas(ii));

        ixx = sigmas(ii)^2*ixx;
        ixy = sigmas(ii)^2*ixy;
        iyy = sigmas(ii)^2*iyy;

        [lambda2, lambda1] = eig_image(ixx, ixy, iyy);

        % Compute some similarity measures
        lambda1(lambda1==0) = eps;
```

```
        Rb = (lambda2./lambda1).^2;
        S2 = lambda1.^2 + lambda2.^2;

        % Compute the output image
        imFilt = exp(-Rb/beta) .*(ones(size(im))-exp(-S2/correction));

        if(opts.tubularity.blackwhite)
            imFilt(lambda1 < 0)=0;
        else
            imFilt(lambda1 > 0)=0;
        end

        filtImArr(:,:,ii) = imFilt;
    end

    imFrangi = max(filtImArr, [], 3);
end


function [im_bw, varargout] = doThresh(im, opts)
% doThresh: this function performs image thresholding on a gray-level
% image.
%
% inputs:
%       im: a 2D gray-level image.
%       opts: a matlab structure containing parameters for the thresholding
%             type:
%          -> opts.thresh: otsu/multi-otsu/kittler/adaptive. By default otsu is
used.
%
% output:
%       im_bw: 2D binarized/thresholded image.

    if ~isfield(opts, 'thresh')
       opts.thresh = 'kittler';
    end

    if ~isfield(opts, 'fillSmallHoles')
       opts.fillSmallHoles = true;
    end

    if ~isa(im,'uint8')
        im = im2uint8(im);
    end

    opts.thresh = lower(opts.thresh);

    im_gray = [];
    if strcmpi(opts.thresh, 'otsu')
        level = graythresh(im);
        im_bw = imbinarize(im,level);
    elseif strcmpi(opts.thresh, 'multiotsu')
        thresh = multithresh(im, 3);
        seg_I = imquantize(im, thresh);
        im_bw = seg_I > 1;
    elseif strcmpi(opts.thresh, 'kittler')
        im = im2uint8(im);
        [optThresh, ~ ] = kittler( im );
        im_bw       = zeros(size(im));
        im_bw(im > optThresh) = 1;
        im_gray     = im;
        im_gray(im < optThresh) = 0;
```

```
    elseif strcmpi(opts.thresh, 'adaptive')
        im = im2uint8(im);
        T = adaptthresh(im, 0.5);
        im_bw = imbinarize(im,T);
    else
        level = graythresh(im);
        im_bw = imbinarize(im,level);
    end

    if opts.fillSmallHoles
       filled     = imfill(im_bw, 'holes');
       holes      = filled & ~im_bw;
       bigholes   = bwareaopen(holes, 30);
       smallholes = ~bigholes & holes;
       im_bw      = im_bw | smallholes;
    end

    %# smoothing thresholded boundaries.
    im_bw = imopen(im_bw, ones(2,2));

    varargout{1} = im_gray;
end


function im_clean = doCleaning(im, opts)
% doCleaning: this function post-process a binary image by removing small
%       objects or by filling holes.
%
% inputs:
%       im: a 2D binary input image.
%       opts.cleaning: a matlab structure containing some parameters for the
post-processing.
%       -> opts.cleaning.minAreaPercent: minimal percenta size of the
%                   total image size for an object to not be removed.
%       -> opts.cleaning.minHoleSize: minimum hole size in pixels to be
%                   filled.
%
% output:
%       im_clean: post-processed binary image.

    if ~isfield(opts, 'cleaning')
        opts.cleaning = [];
    end

    if ~isfield(opts.cleaning, 'minAreaPercent')
        opts.cleaning.minAreaPercent = 0.01/100;
    end

    if ~isfield(opts.cleaning, 'minHoleSize')
        opts.cleaning.minHoleSize = 0;
    end

    bw          = logical(im);
    stats       = regionprops(bw,'area','Perimeter');

    cc          = bwconncomp(bw, 8);
    L           = labelmatrix(cc);

    allAreas    = [stats.Area];

    minArea     = opts.cleaning.minAreaPercent * numel(im);
```

```
    keepMask    = allAreas > minArea;

    bwSmallSegs = ismember(L, find(keepMask));

    leftIdx     = unique(L(:,1));   leftIdx(leftIdx==0) = [];
    rightIdx    = unique(L(:,end)); rightIdx(rightIdx==0) = [];
    topIdx      = unique(L(1,:));   topIdx(topIdx==0) = [];
    bottomIdx   = unique(L(end,:)); bottomIdx(bottomIdx==0) = [];

    bwSmallSegs(ismember(L, leftIdx)) = 1;
    bwSmallSegs(ismember(L, rightIdx)) = 1;
    bwSmallSegs(ismember(L, topIdx)) = 1;
    bwSmallSegs(ismember(L, bottomIdx)) = 1;

    im_clean      = (bw & bwSmallSegs);

    if opts.cleaning.minHoleSize
        minimum_hole_size    = opts.cleaning.minHoleSize;
        allfilled            = imfill(im_clean, 'holes');
        allholes             = allfilled & ~im_clean;
        threshold_holes      = bwareaopen(allholes, minimum_hole_size);
        small_holes          = allholes & ~threshold_holes;
        im_clean               = im_clean | small_holes;
    end

end

function [im_skel, im_branches, varargout] = doSkeleton(im, opts)
% doSkeleton: this function extracts the skeleton from a binary input
% image. Short ramifications can also be pruned.
%
% inputs:
%       im: a 2D binary image input image containing vessels as foreground
objects.
%       opts: a matlab structure containing the parameters for the ramification-
prunning:
%           -> opts.skeleton.cleanSpur: 1 to prune ramifications.
%           -> opts.skeleton.spurLength: length of ramifications to be pruned in
pixel units.
%
% output:
%       im_skel: 2D binary output image with extracted skeleton.
%       im_branches: binary output image with branches as white pixels.

    if ~isfield(opts, 'skeleton')
        opts.skeleton = [];
    end

    if  ~isfield(opts.skeleton, 'cleanSpur')
        opts.skeleton.cleanSpur = 1;
    end

    im_skel = bwmorph(im, 'thin', 'inf');

    im_branches = bwmorph(im_skel, 'branchpoints');

    if opts.skeleton.cleanSpur == 1
        [im_skel_d, ~]  = getPrunnedBranches(im_skel, opts);
        im_branches_d   = bwmorph(im_skel_d, 'branchpoints');
    else
        im_skel_d = im_skel;
        im_branches_d = im_branches;
```

```
    end

    varargout{1}  = im_skel_d;
    varargout{2}  = im_branches_d;
end

function [skelD, im_branches] = getPrunnedBranches(im_skel, opts)

    if ~isfield(opts.skeleton, 'spurLength')
        opts.skeleton.spurLength = numel(im_skel);
    end

    B = bwmorph(im_skel, 'branchpoints');
    E = bwmorph(im_skel, 'endpoints');

    [rows,cols] = find(E);
    B_loc = (B);

    Dmask = false(size(im_skel));
    for k = 1:numel(cols)
        D = bwdistgeodesic(im_skel,cols(k),rows(k));
        distanceToBranchPt = min(D(B_loc));

        if ~opts.skeleton.spurLength
            Dmask(D < distanceToBranchPt) = true;
        else
            Dmask(D < distanceToBranchPt & distanceToBranchPt <
opts.skeleton.spurLength) = true;
        end
    end
    skelD = im_skel - Dmask;
    skelD = bwmorph(skelD,'thin');

    [yy,xx] = find(B);
    im_branches = zeros(size(im_skel));
    im_branches(sub2ind(size(im_branches), yy, xx)) = 1;
    im_branches = logical(im_branches);

end
```
