# Supplementary material for: AutoTube: a novel software for the automated morphometric analysis of vascular networks in tissues
Source: Angiogenesis. 2018 Oct 28;22(2):223–36. doi: 10.1007/s10456-018-9652-3 (PMC6475513; doi:10.1007/s10456-018-9652-3)
Supplement: Supplementary file 2 — Supplementary material 2 (PDF 16011 KB) [file 10456_2018_9652_MOESM2_ESM.pdf]

AutoTube: a novel software for the automated morphometric analysis of vascular networks in tissues

Javier Montoya Zegarra[2¶], Erica Russo[1¶], Peter Runge[1], Maria Jadhav[1], Ann-Helen Willrodt[1], Szymon Stoma[2], Simon F. Nørrelykke[2], Michael Detmar[1] and Cornelia Halin[1]

[1] Institute of Pharmaceutical Sciences, ETH Zurich, Vladimir-Prelog-Weg 1-5/10, 8093 Zürich, Switzerland
[2] Scientific Center for Optical and Electron Microscopy (ScopeM), ETH Zurich, Wolfgang-Pauli-Str. 14, 8093 Zürich, Switzerland
¶ these authors contributed equally to this work

Corresponding author:
Cornelia Halin, Ph.D.
e-mail: cornelia.halin@pharma.ethz.ch
phone: +41 44 633 29 62

Supplementary Figure 1

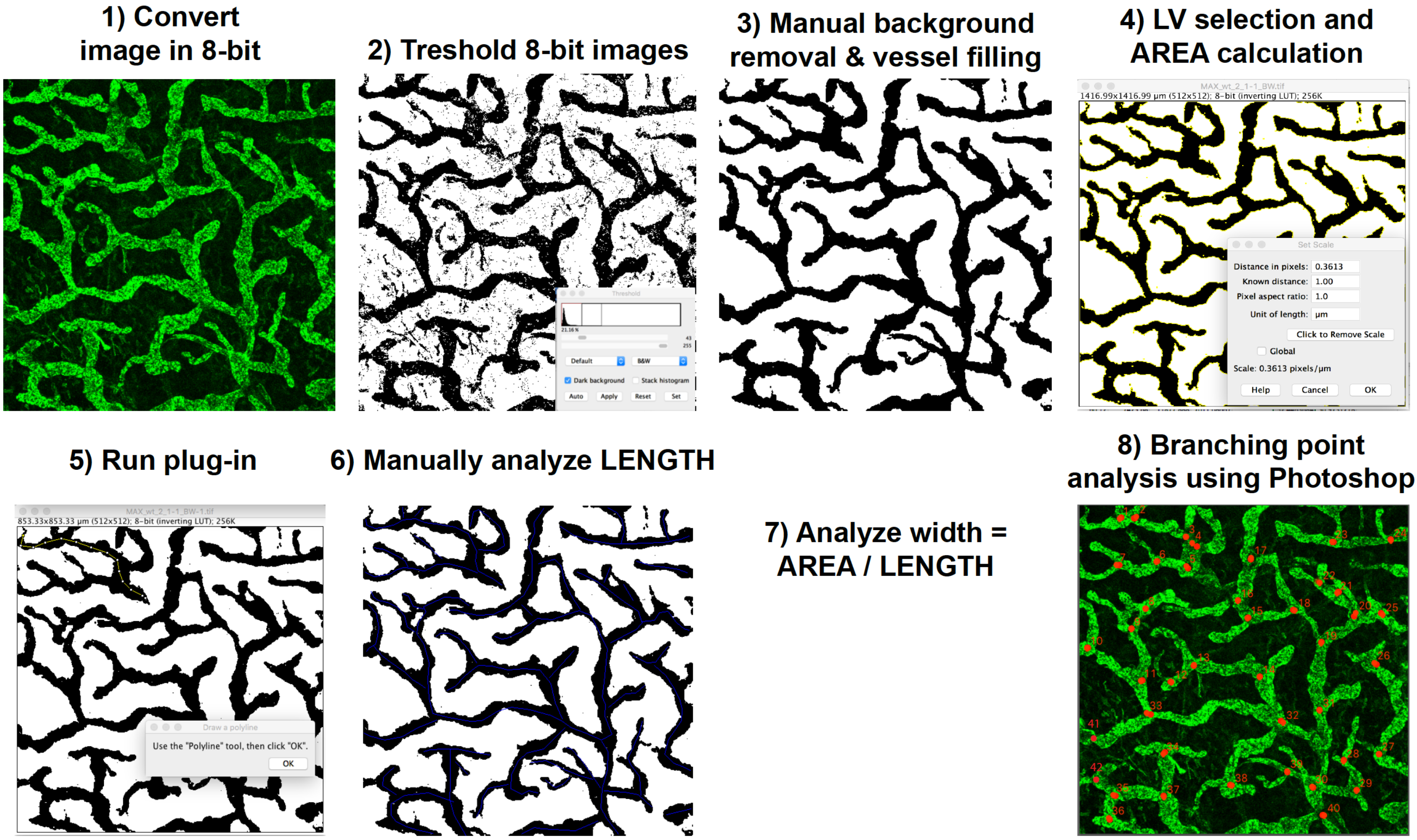

Supplementary Figure 1. Manual analysis protocol. (1) RGB fluorescent images are converted into 8-bit pictures. **(2)** Thresholding is applied to the images; inverted LUT (lookup table): 0 corresponds to white background and 255 to black vessel. **(3)** The background is manually cleaned and the vessels are filled to counteract patchy staining. **(4)** The black lymphatic vessels (LV) are selected. The pixel-μm conversion is set and area calculated **(5-6)** A self-made plugin is run in Fiji and the skeleton length is manually calculated using the polyline tool. **(7)** The width is calculated as the fraction between area and total length. **(8)** In the last step, branching points are manually counted using Photoshop or Fiji.

Supplementary Figure 2

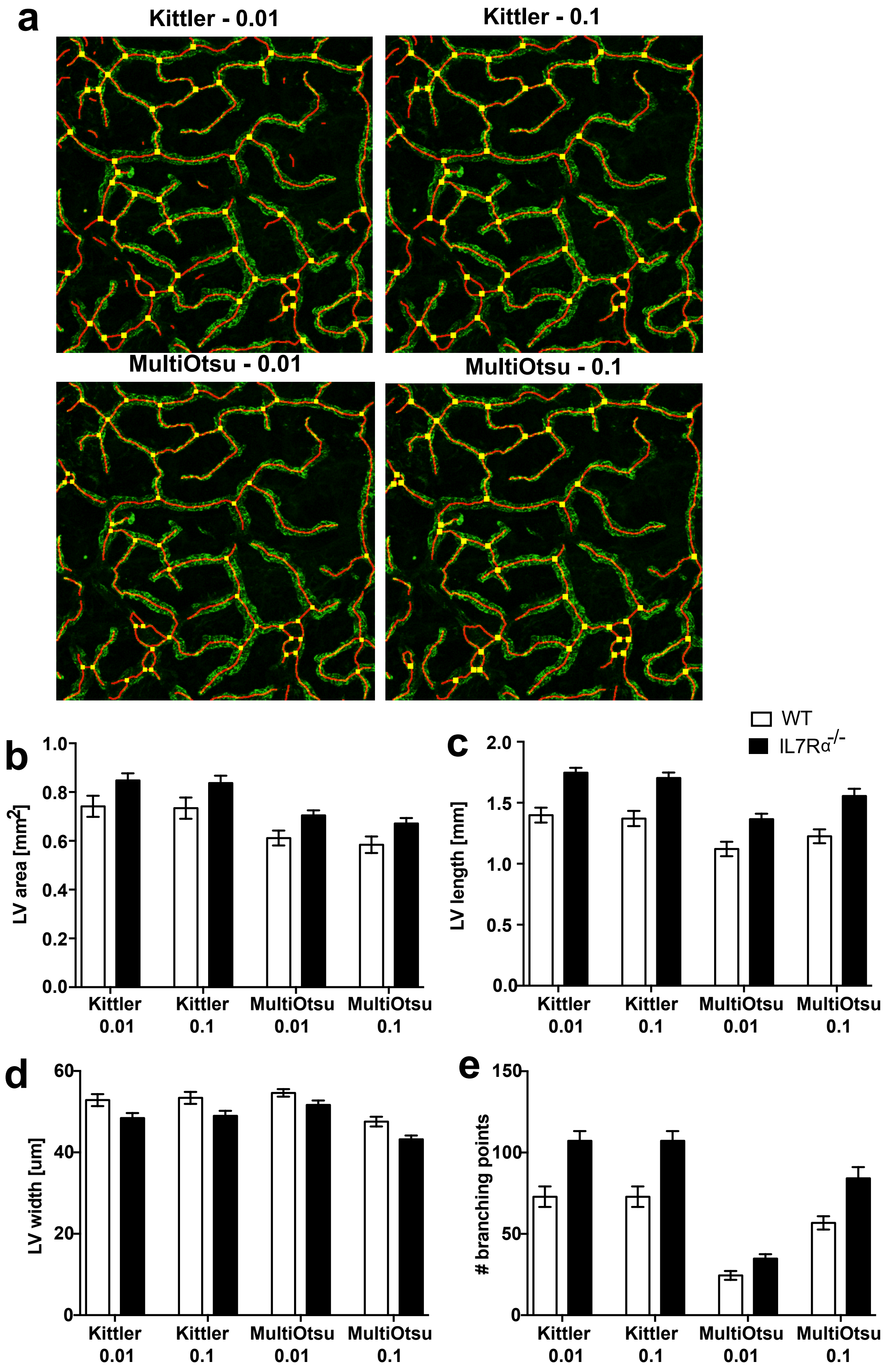

Supplementary Figure 2. Parameter evaluation for lymphatic vessels in WT and IL-7Rα$^{-/-}$ mice using different thresholding methods. (a-e) Comparison between Kittler and Multi-Otsu thresholding methods coupled with the influence of small detected vessels removal. **(a)** Lymphatic vessels (LVs) were detected in an ear skin whole-mount by staining with anti-LYVE-1 antibody. **(b-e)** Results show that the measured morphometric properties (LV area, length, width, and number of branching points) are rather stable.

Supplementary Figure 3

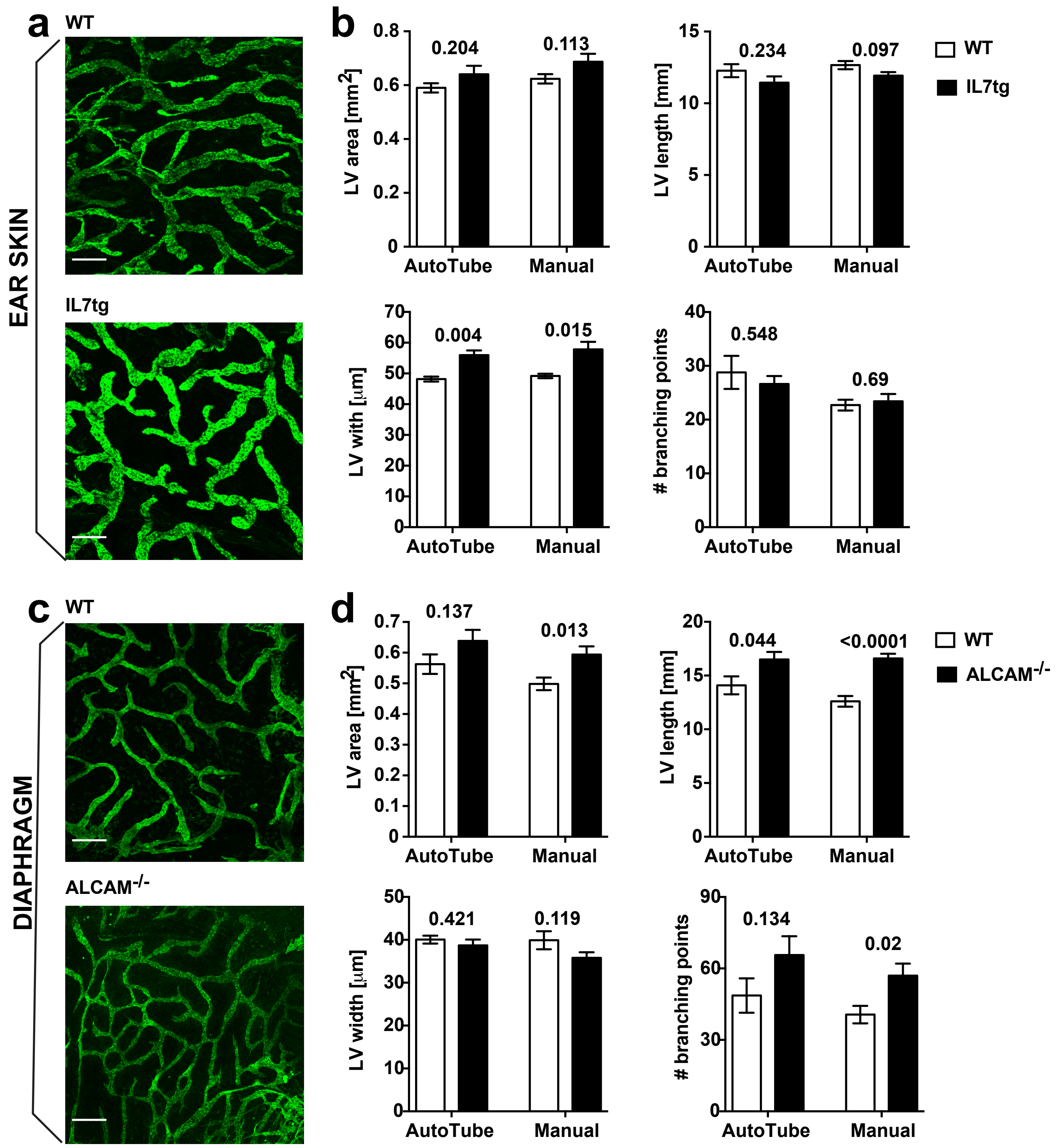

Supplementary Figure 3. AutoTube-based and manual analysis of the LYVE-1^{+} lymphatic vasculature in tissue whole-mounts prepared from the murine ear skin (WT and IL7tg) and diaphragm (WT and ALCAM-/-). Parameters analyzed include the entire area covered by lymphatic vessels (LV area), the absolute length of the network (LV length), the average vessel width (LV width) and the number of branching points (# branching points) per picture analyzed. **(a)** Representative images of LYVE-1^{+} lymphatic vessels (LVs) in the ear skin of WT and IL7tg mice. Scale bar = 100µm **(b)** Results from manual and from AutoTube-based quantification of lymphatic vessels in WT and IL7tg ear skin. **(c)** Representative images of LYVE-1^{+} lymphatic vessels in the diaphragm of P6 WT and ALCAM-/- pups. Scale bar = 100µm **(d)** Results from manual and AutoTube-based quantification of lymphatic vessels in P6 WT and ALCAM-/- pups. The data shown represent the mean ± SEM; statistical analysis was performed with Student's t-test; ns=not significant for p>0.25; n = 5-7 mice each group (3 pictures/mouse).

Supplementary Figure 4

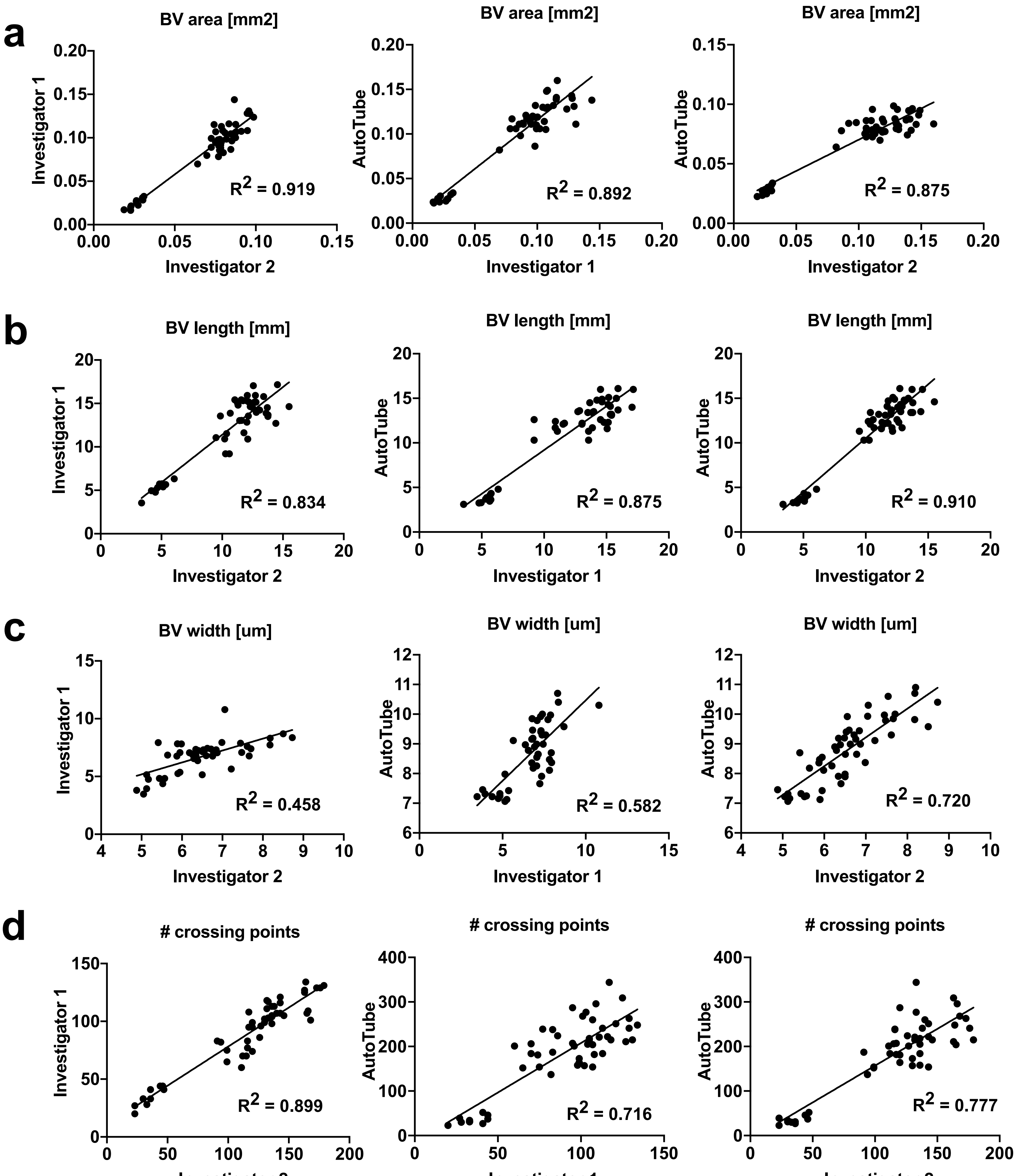

Supplementary Figure 4. Analysis of the correlation between the two manual and the automated quantification of all blood vessel parameters analyzed in Fig 5b (diaphragm and ear skin). (a) blood vessel area (BV area) **(b)** blood vessel length (BV length) **(c)** blood vessel width (BV width) and **(d)** # crossing points. n = 51 images in total from diaphragms and ear skins are shown.

Supplementary Figure 5

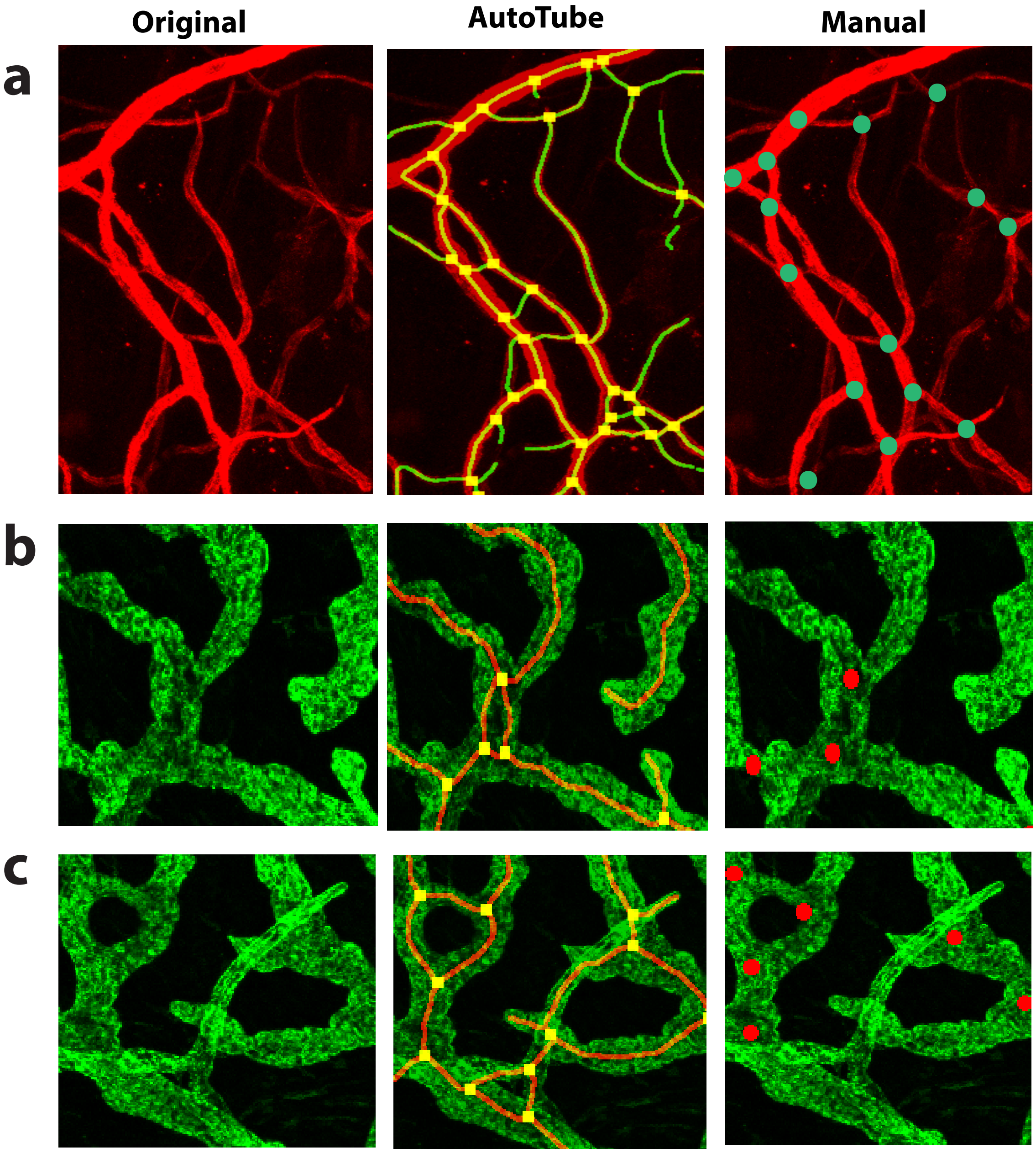

Supplementary Figure 5. Impact of staining quality and presence of overlapping vessel branches on the blood vessels crossing point / lymphatic vessel branching point analysis. (a) blood vessels detected in an ear skin whole-mount by staining with anti-MECA-32 antibody (left). Crossing points were determined by AutoTube-based (middle) or by manual (right) analysis. More true-positive crossing points were detected by Auto-Tube, likely because the manual analysis failed to detect crossing points formed by faint vessels or crossing points in immediate proximity to each. **(b, c)** lymphatic vessels detected in an ear skin whole-mount by staining with anti-LYVE-1 antibody (images on left). Branching points were determined by AutoTube-based (middle) or manual (right) analysis. **(b)** Example of a false detection of lymphatic vessel branching points by AutoTube, caused by a vessel branch in the foreground that appears to overlap with other vessels parts in the background. **(c)** Example a heterogeneously stained LYVE-1^{+} vessel area, which AutoTube interprets as two separate vessel segments and additional, false-positive lymphatic vessel branching points.
